# Molecular Recognition of Disaccharides in Water: Preorganized Macrocyclic or Adaptive Acyclic?

**DOI:** 10.1002/chem.202101238

**Published:** 2021-06-01

**Authors:** Oscar Francesconi, Francesco Milanesi, Cristina Nativi, Stefano Roelens

**Affiliations:** ^1^ Department of Chemistry “Ugo Schiff” and INSTM University of Florence Polo Scientifico e Tecnologico 50019 Sesto Fiorentino, Firenze Italy; ^2^ Magnetic Resonance Center CERM Via L. Sacconi 6 50019 Sesto Fiorentino, Firenze Italy

**Keywords:** carbohydrates, chitobiose, hydrogen bonds, molecular recognition, receptors

## Abstract

When facing the dilemma of following a preorganized or adaptive design approach in conceiving the architecture of new biomimetic receptors for carbohydrates, shape‐persistent macrocyclic structures were most often chosen to achieve effective recognition of neutral saccharides in water. In contrast, acyclic architectures have seldom been explored, even though potentially simpler and more easily accessible. In this work, comparison of the binding properties of two structurally related diaminocarbazolic receptors, featuring a macrocyclic and an acyclic tweezer‐shaped architecture, highlighted the advantages provided by the acyclic receptor in terms of selectivity in the recognition of 1,4‐disaccharides of biological interest. Selective recognition of GlcNAc_2_, the core fragment of N‐glycans exposed on the surface of enveloped viruses, stands as an emblematic example. NMR spectroscopic data and molecular modeling calculations were used to ascertain the differences in binding mode and to shed light on the origin of recognition efficacy and selectivity.

## Graphical abstract

**Adaptive to be selective**: Comparison of the binding properties of two structurally related biomimetic receptors toward disaccharides sheds light on the differences between a rigid macrocyclic architecture and a flexible tweezer‐shaped structure showing, counterintuitively, that the adaptive structure can be advantageous in terms of selectivity. NMR spectroscopy and molecular modeling calculations were used to provide a 3D description of the receptor‐disaccharide complexes in solution and explaining the origin of the observed selectivity.

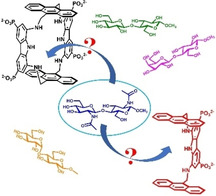

## Introduction

Among the plethora of biologically relevant oligosaccharides, those connected by a glycosidic 1,4‐linkage are plentiful in nature. Lactose and maltose are two of the most common 1,4‐disaccharides, whereas cellulose and chitin, constituted by repeating units of cellobiose (Glc_2_, CeB) and of *N*,*N’*‐diacetylchitobiose (GlcNAc_2_), respectively, both connected by 1,4‐glycosidic linkages, are among the most abundant biopolymers in nature.[^1^, ^2^] Glycosidic 1,4‐linkages are also very common in glycan structures. For example, the disaccharide GlcNAc_2_ is a part of the GlcNAc_2_Man_3_ fragment, highly conserved in the core of N‐glycans exposed on the surface of enveloped viruses, some of which are particularly hazardous for human health, including, among others, coronaviruses and retroviruses.[^3^, ^4^]

Molecular recognition of disaccharides of biomedical relevance by biomimetic receptors in physiological media represents a major challenge of current research,[^5^, ^6^] because selective recognition of neutral saccharides in water must cope with a highly competitive solvent.^[7]^ Nevertheless, in the last few years significant steps forward have been made by developing biomimetic receptors based on rigid macrocyclic architectures.^[8]^

Although this approach has been quite successful for the recognition of several mono‐ and oligosaccharides, it is hampered by lengthy multistep syntheses of low overall yields, due to the critical macrocyclization step.^[9]^ On the other hand, examples of effective recognition of neutral saccharides in water by acyclic receptors are extremely rare in the literature,[^10^, ^11^] even though acyclic flexible architectures can take advantage of being more easily adaptable to the guest, while featuring simpler structures suitable for further optimization.

**Figure 1:**
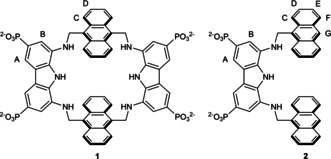
Structure of receptors **1** and **2** with proton labeling.

We have recently reported two biomimetic receptors (**1** ^[12]^ and **2**,^[13]^ Figure 1) effectively recognizing carbohydrates in water. The two receptors share a common tridentate diaminocarbazole hydrogen binding motif, equipped with phosphonate hydrosolubilizing groups, and two anthracene groups, providing extended CH‐π interactions with the saccharidic backbone.^[14]^ Receptor **1** features a preorganized macrocyclic structure possessing a hydrophobic cavity lined with H‐bonding groups, whereas receptor **2** possesses a flexible, acyclic, tweezer‐shaped architecture featuring analogous binding motifs. Receptor **1**, easily available in six steps with 30 % overall yield, effectively binds monosaccharides in water, selectively recognizing the beta anomer of glucose with a 1.3 mM affinity (expressed as intrinsic median binding concentrations, BC050 ), and the α anomers of glucose, galactose, and fucose with affinities of 3.12, 1.19 and 360 μM, respectively.^[12]^ Although extensively investigated toward monosaccharides, the binding properties of receptor **1** toward disaccharides were not yet explored. On the other hand, receptor **2**, which has been shown to effectively recognize 1,4‐disaccharides, with a marked affinity (160 μM) and selectivity for the methyl β‐glycoside of GlcNAc_2_,^[13a]^ did not bind to monosaccharides at all.

In order to assess the role of the architecture in saccharide recognition, in this work we investigated the binding affinities of receptor **1** toward the set of glucose‐containing disaccharides used to test receptor **2**, to compare their binding properties and ascertain the effect of macrocyclic (preorganized) versus acyclic (adaptive) structures on recognition ability.^[15]^ NMR‐based molecular modeling calculations were used to give a three‐dimensional description of the complexes of the two receptors with a common guest, which revealed the substantial role of CH‐π interactions.

## Results and Discussion

**Figure 2:**
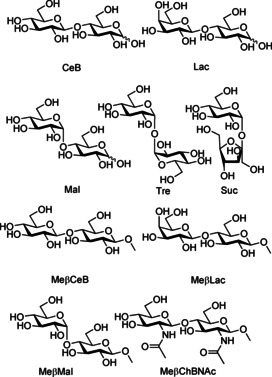
Structures of the investigated disaccharides and their abbreviations.

In a preliminary screening by ^1^H NMR spectroscopy, the binding ability of **1** was tested toward a set of disaccharides constituted by at least one glucose unit, for which the receptor showed good affinities, including cellobiose (CeB), lactose (Lac), maltose (Mal), trehalose (Tre), and sucrose (Suc; Figure 2). Binding ability was qualitatively evaluated by monitoring the shifts of the proton signals of the sugar upon addition of an equimolar amount of **1**. Although for Suc and Tre no variations were observed, a marked upfield shift was detected for CeB, Mal and Lac, reasonably due to the shielding effect of the anthracene moieties in the binding cavity, larger for the β than for the α anomers. A concomitant broadening of signals, larger for the β anomers, indicated slow chemical exchange, most likely due to strong binding (Figures S1–S3 in Supporting Information).

For a quantitative determination of the binding ability of **1**, ^1^H NMR titrations of methyl‐β‐glycosides of cellobiose (MeβCeB), lactose (MeβLac), and maltose (MeβMal) were carried out in D_2_O (pD 7.4) at 298 K, additionally including MeβGlcNAc_2_, for which receptor **2** showed high affinity (Figure 2). To avoid ambiguities in the definition of the binding model, the cumulative association constants reported in Table 1 were obtained by the simultaneous fit of all available signals from two independent titrations, run at different reactant concentrations. Because multiple complex species were found for all systems, the overall affinities reported in Table 1 were determined by the intrinsic median binding concentration parameter (BC050 ),^[16]^ which was calculated from the measured binding constants. ^1^H NMR titrations with MeβMal were also duplicated at pD 11 (Table S1) and fitted to the association model obtained at pD 7.4. While protonation of the aromatic amino groups is not expected in the investigated range of pD, the degree of protonation of the phosphonate groups does not affect the binding ability of receptor **1**, as previously observed for binding to monosaccharides^[12]^ and confirmed by the comparable affinities obtained at different pD values.

**Table 1:** Cumulative formation constants (log *β*_n_)^[a]^ and intrinsic median binding concentration (BC050 , [mM])^[b]^ for receptor to glycoside (R : G) complexes of **1** and **2** with methyl glycosides, measured at 298 K from NMR data in D_2_O at pD 7.4.^[c]^

Receptor		**1**		**2**	
Glycoside	R : G	log *β*	BC050	log *β*	BC050
MeβCeB	1 : 1	3.27±0.02	1.15±0.04	2.53±0.07	0.94±0.10
	1 : 2	4.92±0.03			
	2 : 1	6.81±0.02		6.33±0.06	
	2 : 2	8.91±0.06			
MeβMal	1 : 1	3.29±0.05	1.06±0.07	2.27±0.01	31.0±4.4
	1 : 2	4.77±0.05			
	2 : 1	6.82±0.04			
	2 : 2	9.21±0.09			
MeβLac	1 : 1	3.19±0.01	1.43±0.05	2.27±0.02	30.8±4.7
	1 : 2	4.42±0.02			
	2 : 1	6.22±0.04			
	2 : 2	8.04±0.13			
MeβGlcNAc_2_	1 : 1	n.d.^[d]^		3.55±0.04	0.16±0.01
	2 : 1			7.35±0.09

[a] Formation constants were obtained by nonlinear least‐square regression analysis of NMR data. [b] Calculated from the log *β* values using the “BC50 Calculator” program.^[16]^ [c] Receptor dimerization constants at pH 7.4 (**1**: log *β*_dim_=3.84±0.20; **2**: log *β*_dim_=2.65±0.07) were set invariant in the nonlinear regression analysis of NMR data. [d] not detectable.

As with monosaccharides, Table 1 shows multiple binding constants for receptor **1** with disaccharides. Strong self‐association, with a dimerization constant of log *β*_dim_=3.84±0.20, favors complex species in which the receptor is dimeric, featuring two binding cavities and giving rise to complexes with stoichiometries higher than 1 : 1. Results show that receptor **1** effectively binds to MeβCeB, MeβMal, and MeβLac with good affinities, though with lack of selectivity, but does not recognize MeβGlcNAc_2_, for which no significant variations of chemical shifts were detected (Figure S8). Thus, receptor **1** can distinguish 1–4 from 1–1’ disaccharides (Suc/Tre), which are not bound at all, and MeβCeB from the N‐acetylated amino‐analogue MeβGlcNAc_2_, but cannot discriminate among glucose containing 1–4 disaccharides, proving to be insensitive to the configuration of the anomeric linkage (MeβCeB/ MeβMal) and to the presence of axial substituents (MeβLac). Surprisingly, these 1–4 disaccharides are bound with an affinity very close to that previously observed for MeβGlc, indicating lack of selectivity between mono‐ and disaccharides.

Comparison of binding properties between receptors **1** and **2**, as obtained by ^1^H NMR titrations, quantifies the selectivity advantage achieved through the adaptive architecture. Indeed, in contrast to **1**, receptor **2** not only strongly binds to MeβGlcNAc_2_, but also discriminates among the investigated glucose containing 1,4‐disaccharides. Receptor **2** shows preference for the all‐equatorial MeβCeB, which is bound with an affinity very close to that observed for **1**, whereas MeβMal and MeβLac are bound with an affinity more than one order of magnitude smaller.

To shed light on the origin of such unexpected difference between **1** and **2**, a description of the binding mode characterizing the receptor‐disaccharide complexes in solution was attempted by combining NMR techniques with molecular modeling calculations, following the approach previously adopted to study the complex between MeβGlcNAc_2_ and receptor **2**, which provided an informative picture of the interaction.^[13a]^ Because MeβGlcNAc_2_ did not bind to **1**, the investigation was carried out on MeβCeB, MeβMal and MeβLac.

**Figure 3:**
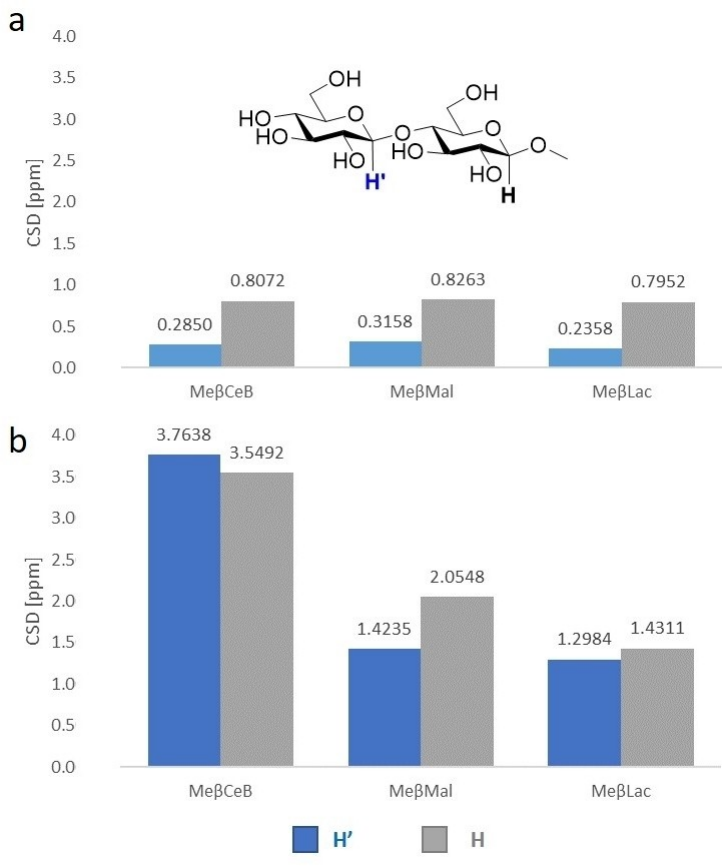
Plot of the chemical shift differences (CSD, ppm) between the free and bound states of the anomeric H and H’ protons for MeβCeB (shown with proton labeling), MeβMal and MeβLac when bound to a) **1** and b) **2** in 1 : 1 complexes in D_2_O (pD 7.4) at *T*=298 K.

Chemical shift variations of the anomeric protons H‐1 and H’‐1 upon formation of the 1 : 1 complex with receptor **1**, as calculated by nonlinear regression analysis of titration data, showed an upfield shift for all three disaccharides caused by the aromatic shielding effect (Figure 3a and Table S2), which is more pronounced on the H proton of the methyl‐β‐glucoside unit (Δ*δ*=0.80–0.82) than on the H’‐1 proton (Δ*δ*=0.24–0.32), suggesting a closer contact of the former to the aromatic moieties. On the other hand, from the analysis of chemical shift variations in the 1 : 1 complexes with receptor **2** (Figure 3b and Table S3), a stronger shielding effect is clearly apparent, more marked for MeβCeB, indicating a closer proximity of the entire disaccharide to the aromatic rings. This evidence suggests that **2** can adapt better than **1** to the disaccharidic guests, showing preference for the all‐equatorial MeβCeB. The chemical shift differences (CSDs) for the H‐1 and H’‐1 protons of the latter are very similar, suggesting a fit of the entire disaccharide into the cleft of the receptor. The CSDs of MeβMal and MeβLac, significantly smaller and with the H‐1 CSD predominant, suggest a less comfortable fit in the cleft, in agreement with the corresponding lower affinities.

The complexes of receptor **1** and **2** with MeβCeB were then selected as representative examples, and their binding modes were studied by NOESY spectroscopy at pD 11, a medium in which the receptors are fully deprotonated species. From NOESY spectra run on the 1 : 1 mixture of **1** and MeβCeB, a strong intramolecular NOE contact was found between the H’‐1 and the H‐4 protons (Figure S10), suggesting that in the complex the disaccharide is in the conformation usually found in solution. Unambiguous intermolecular NOE contacts were also identified (Figures S11 and S12), the strongest of which were those between the OCH_3_ protons and both the H−C and H−D protons of the anthracene ring (Figure 1), and between the H’‐1/H’‐5 protons and the H−D protons.

NOESY spectra performed on an equimolar mixture of **2** and MeβCeB showed unambiguous intermolecular NOE contacts between both saccharidic units of MeβCeB and the anthracene protons of **2** (Figure S15). The NOESY map shows a strong NOE cross peak between H‐2 and H−C, and a NOE contact of H’‐2 with the H−F located on the opposite side of the anthracene ring. Moreover, the OCH_3_ protons show NOE contacts with the H−C, H−D and H−E protons.

**Figure 4:**
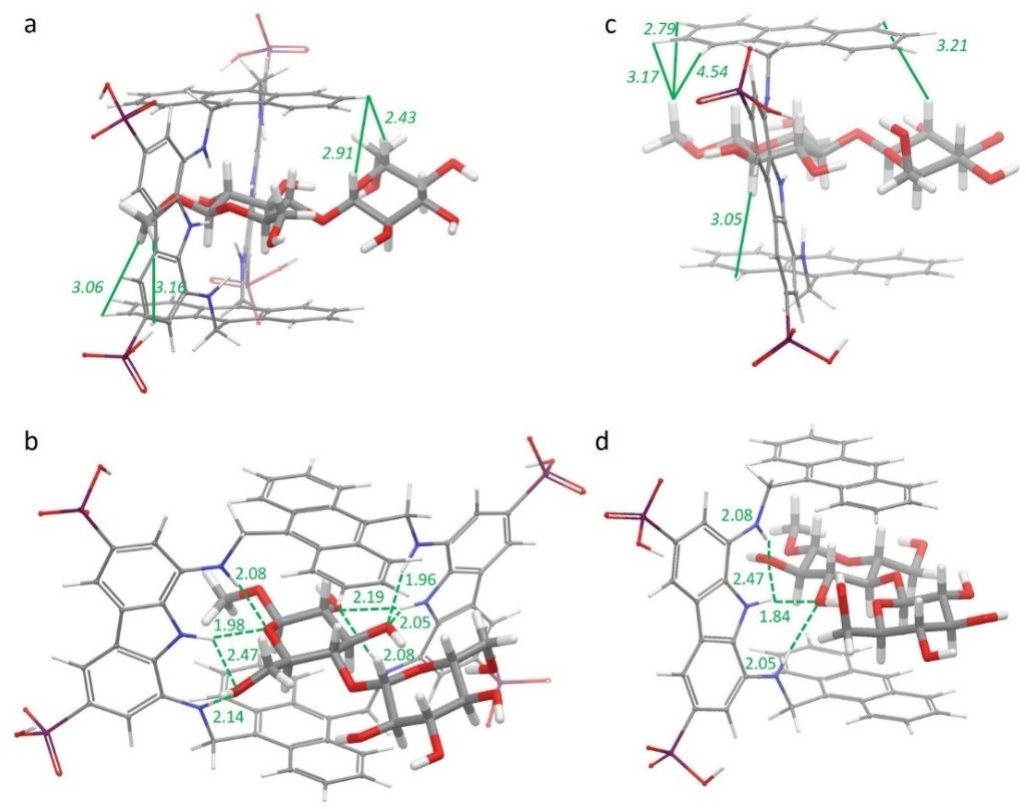
Global minimum structures of the a) and b) **1**⋅MeβCeB and c) and d) **2**⋅MeβCeB complexes in two different projections. The strongest intermolecular NOEs found between a) **1** and MeβCeB and c) **2** and MeβCeB are indicated as solid lines, with the corresponding distances [Å] calculated for the lowest energy conformer. Intermolecular hydrogen‐bonding interactions found in the calculated structures are indicated as dashed lines in (b) and (d), together with the corresponding oxygen/hydrogen distances [Å].

Based on NOESY NMR evidence, molecular mechanics calculations were carried out on the 1 : 1 complex of **1** with MeβCeB, on the assumption that, although prevalently dimeric, the receptor would feature two independent binding sites. A conformational search, using a well‐tested unconstrained molecular mechanics protocol,^[17]^ returned a family of conformers within 5.19 kJ mol^−1^ from the global minimum that was in very good agreement with NMR spectroscopic data. The minimum energy structure depicted in Figure 4a and b shows MeβCeB partially located inside the receptor cavity, with the methyl glycoside unit nested inside the cavity and the other unit protruding outward, in a geometry that agrees with the strongest NOE contacts observed in NOESY maps (Table S4) and with the shift differences observed from titration experiments (Figure4a). All O⋅⋅⋅H interatomic distances shorter than the sum of the van der Waals radii and compliant with hydrogen bonding criteria were calculated from the above model, and several hydrogen‐bonding interactions were found involving the methyl glucoside unit exclusively (Figure 4b). Additional contribution to binding is provided by several CH‐π interactions showing short distances, established between the methyl glucoside unit and the anthracene rings (Table S6).

The binding geometry obtained from calculations supports the observed affinities. Indeed, because the disaccharide is bound through the methyl glycoside unit exclusively, lack of selectivity among the investigated set of disaccharides can be easily anticipated, irrespective of the α/β glycosidic linkage to the second unit. This evidence also explains the closely similar affinities observed between the disaccharides and the monosaccharide MeβGlc. Likewise, the 1–1’ disaccharides, featuring a bulky substituent in place of the methyl group, and MeβGlcNAc_2_ featuring the N‐acetyl groups, can hardly fit into the receptor cavity. Thus, despite the good affinities observed, lack of selectivity between glucose containing 1,4‐disaccharides can be ascribed to the size of the macrocyclic cavity, unable to accommodate the entire disaccharide.

The conformational search carried out on the 1 : 1 complex between **2** and MeβCeB resulted in a single family of minimum energy conformers within 8.73 kJ mol^−1^ from the global minimum. The minimum energy structure depicted in Figure 4c shows the MeβCeB entirely located inside the binding cleft between the two anthracene faces, in a geometry closely similar to that previously observed in the complex with MeβGlcNAc_2_,^[13a]^ and in agreement with the proximities inferred by strong NOE contacts (Table S5).

Hydrogen bonding interactions could be calculated from the above model (Figure 4d) and, analogously to **1**, four hydrogen bonds were found between the diaminocarbazole unit and MeβCeB. However, in contrast to **1**, a significant enhancement to binding could result from the extensive network CH‐π interactions that can be established between the axial protons of both the saccharidic units and the anthracenes (Table S6).

The above three‐dimensional descriptions clearly show that the acyclic structure of **2** can adapt to the disaccharidic guest better than the macrocyclic structure of **1**, giving rise to increased affinity despite the lack of a hydrogen‐bonding unit. The evidence indicates that the latter is effectively compensated for by a tighter fit and by extensive CH‐π interactions. Such compensation is not fully achieved with MeβMal and MeβLac because axial substituents hamper a tight fit into the cleft, causing a drop in affinity. In contrast, the lack of preorganization and absence of a hydrogen‐bonding unit cause a severe drop in the affinity of **2** for monosaccharides, which is not compensated for by additional interactions; this results in undetectable binding. Thus, macrocyclic receptor **1** appears to be well preorganized for binding a monosaccharidic but not a disaccharidic guest, whereas acyclic receptor **2** can take advantage of its adaptive structure to establish more extensive attractive interactions with respect to its macrocyclic counterpart.

The enhanced binding of **2** to MeβGlcNAc_2_ compared to MeβCeB could be explained by the additional hydrogen bonding and CH‐π interactions involving the N‐acetyl group that the former can establish with the receptor.

## Conclusion

Together, the results presented demonstrate that a flexible acyclic structure can be an effective alternative to the widely studied macrocyclic architectures for the molecular recognition of neutral disaccharides in water, provided that a suitably designed combination of hydrogen bonding and CH‐π interactions can be established with the saccharidic guest. The structurally simple tweezer‐shaped receptor **2** presents significant advantages over its macrocyclic counterpart **1**, accommodating the disaccharidic guest within the binding cleft and selectively recognizing the methyl‐β‐glycoside of GlcNAc_2_ over a set of monosaccharides and structurally related 1,4 disaccharides. Because of its simple structure, easy synthetic availability, and potential for structural modifications, the tweezer‐shaped architecture of receptor **2** opens the way to the design of acyclic receptors for the recognition of saccharides in water.

## Conflict of interest

The authors declare no conflict of interest.

## Supporting information

As a service to our authors and readers, this journal provides supporting information supplied by the authors. Such materials are peer reviewed and may be re‐organized for online delivery, but are not copy‐edited or typeset. Technical support issues arising from supporting information (other than missing files) should be addressed to the authors.

Supplementary
