# Supplementary material for: Molecular Recognition of Disaccharides in Water: Preorganized Macrocyclic or Adaptive Acyclic?
Source: Chemistry. 2021 Jun 1;27(40):10456–60. doi: 10.1002/chem.202101238 (PMC8361761; doi:10.1002/chem.202101238)
Supplement: Supplementary file 1 — Supplementary [file CHEM-27-10456-s001.pdf]

Chemistry–A European Journal

Supporting Information

Molecular Recognition of Disaccharides in Water: Preorganized Macrocyclic or Adaptive Acyclic?

Oscar Francesconi,* Francesco Milanesi, Cristina Nativi, and Stefano Roelens*

SUPPORTING INFORMATION

**Binding studies**	**p. S2**
**NMR preliminary screening**	**p. S2**
**NMR titrations and data analysis**	**p. S5**
**Structural studies**	**p. S30**
**Chemical shift difference (CSD) analysis**	**p. S30**
**NMR methods**	**p. S31**
**Molecular modeling methods**	**p. S38**
**References**	**p. S41**

Binding studies.

NMR preliminary screening. Preliminary screenings (298 K, 500 MHz) were performed in D_2O at pD 11 in presence of DSS as internal reference. Solution of reducing carbohydrates were prepared in D_2O and kept overnight at room temperature before the screening experiments, to ensure equilibration of the anomers. The spectra of the free sugars at 1 mM concentration were compared to the spectra of the equimolar mixture of sugars with receptor **1** (1 mM each) and chemical shift differences were evaluated.

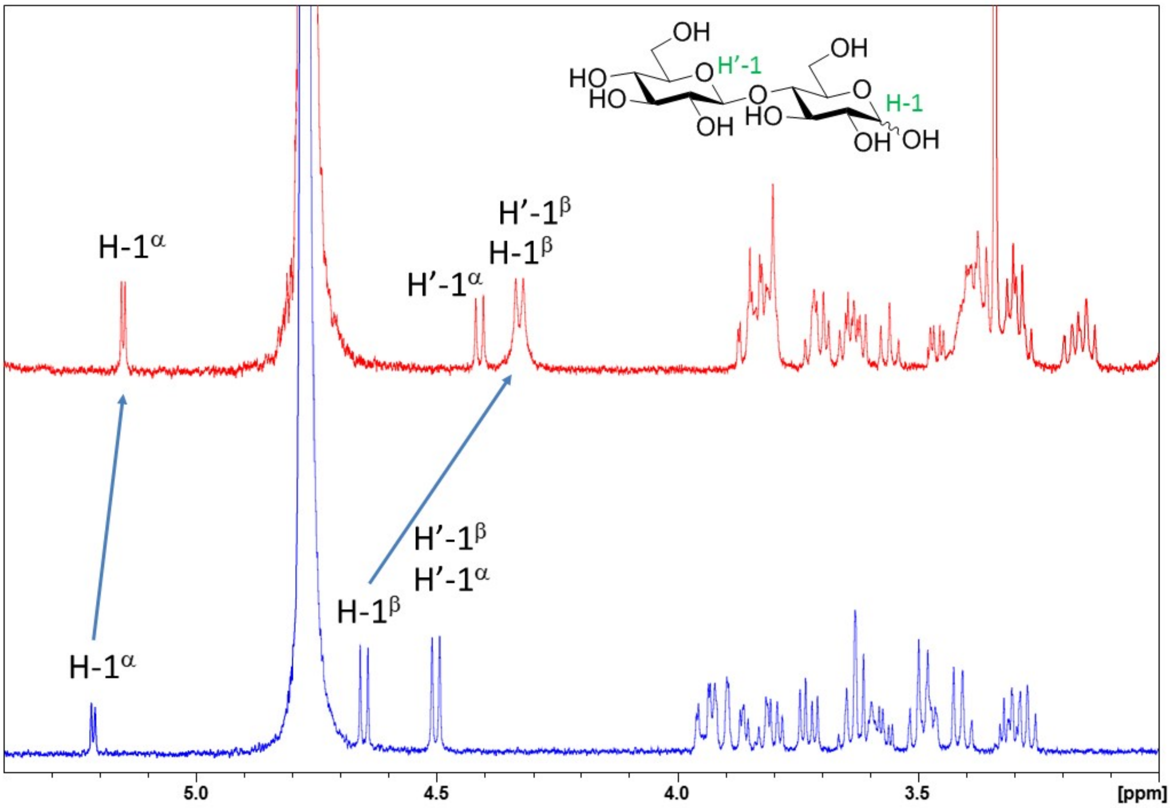

Figure S1. ^{1}H NMR spectra (500 MHz, D_2O) of a 1 mM solution of CeB (bottom) and of an equimolar mixture of CeB and **1** (top, 1 mM each) at pD 11. Variations of the H-1 proton signals are $\Delta\delta = 0.06$ ppm for the α and $\Delta\delta = 0.32$ ppm for the β anomer.

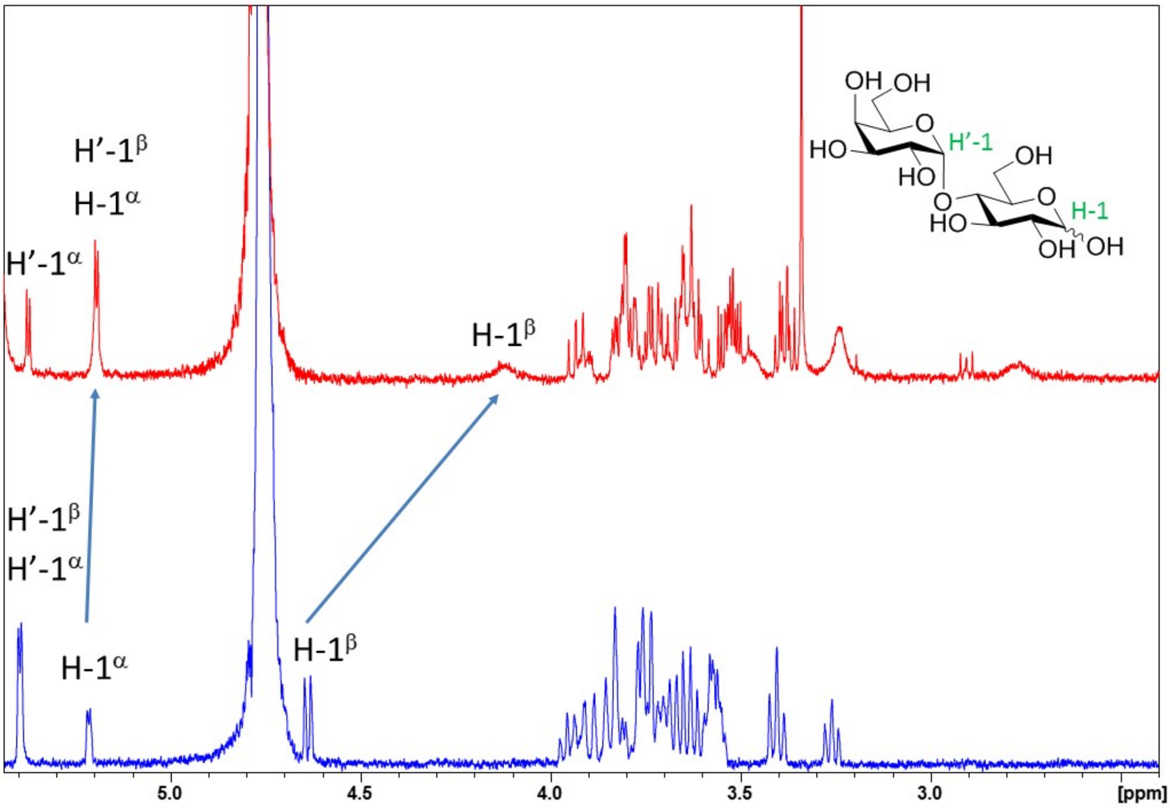

Figure S2. ^{1}H NMR spectra (500 MHz, D_2O) of a 1 mM solution of Mal (bottom) and of an equimolar mixture of Mal and **1** (top, 1 mM each) at pD 11. Variations of the H-1 proton signals are $\Delta\delta = 0.02$ ppm for the α and $\Delta\delta = 0.52$ ppm for the β anomer.

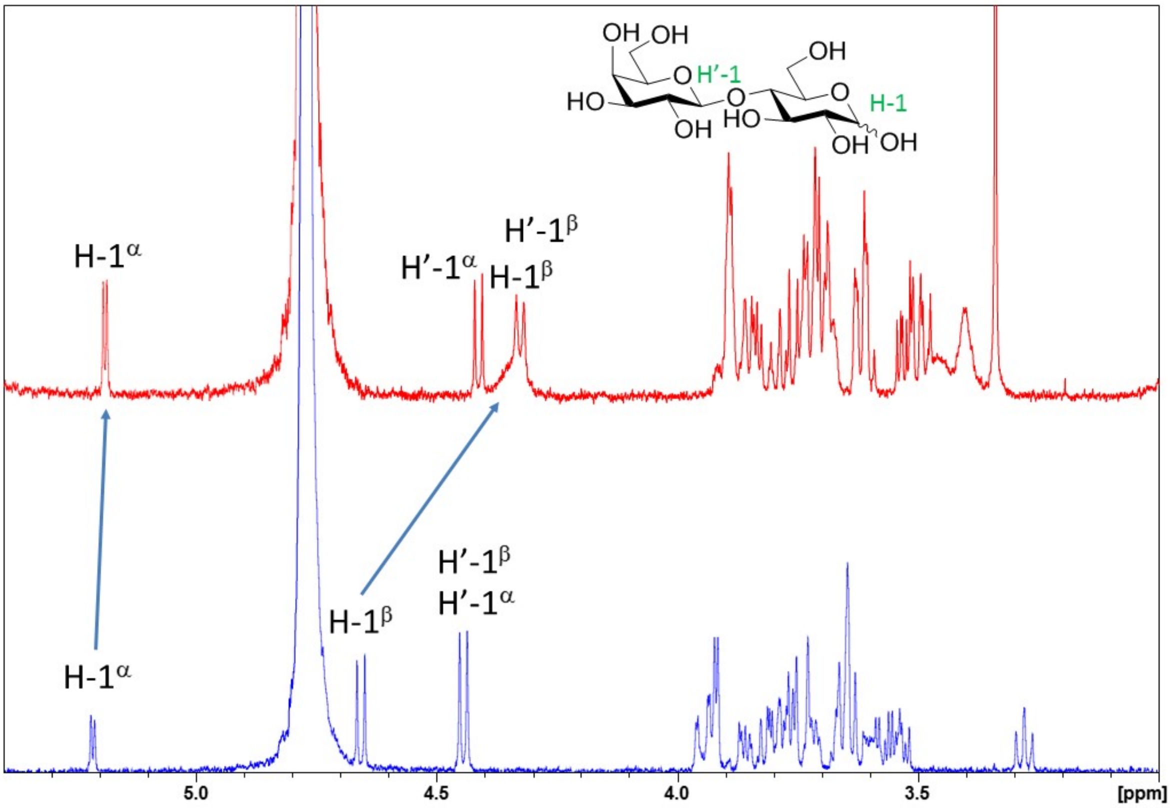

Figure S3. ^{1}H NMR spectra (500 MHz, D_2O) of a 1 mM solution of Lac (bottom) and of an equimolar mixture of Lac and **1** (top, 1 mM each) at pD 11. Variations of the H-1 proton signals are $\Delta\delta = 0.03$ ppm for the α and $\Delta\delta = 0.31$ ppm for the β anomer.

NMR titrations and data analysis. Titrations were performed at 298 K, 500 MHz in 5 mm NMR tubes using microsyringes, following a previously described technique.[S1] Concentration of the receptor was maintained constant during the titrations with glycosides to avoid changes in ionic strength. The stock solutions of **1** was prepared in D_2O adjusting the pD with a diluted NaOH solution in D_2O. A correction factor of + 0.4 was applied to the pH values measured by the pH meter to determine the pD values (pD = pH + 0.4). The alkaline stock solution of **1** was stored during the titrations under nitrogen atmosphere to avoid acid/base reactions with atmospheric CO_2. Following this strategy, constant values of pD were observed during titrations and dilution experiments. DSS was used as internal reference. Dimerization constants of receptor **1** at pD 7.4 and at PD 11, were set invariant in the non-linear regression analysis of receptor-glycosides binding data measured at pD 7.4 and pD 11, respectively. To avoid any ambiguities in the definition of the equilibrium model with receptor **1**, independent titrations were performed at significantly different receptor concentrations and all set of data were simultaneously fitted through a nonlinear least-square regression analysis, including in the fit all the available signals from both reactants at pD 11 and from the glycoside only at pD 7.4. Mathematical analysis of data and graphic presentation of results was performed using the HypNMR 2006 program.[S2] BC_{50} Calculator, the utility program for computing BC_{50}^0,[16] is available for free at the corresponding author's e-mail address. Results pages and Plots of experimental and calculated shifts are reported hereafter.

1 + MeβCeB (D_2O, pD 7.4, 298 K, 500 MHz)

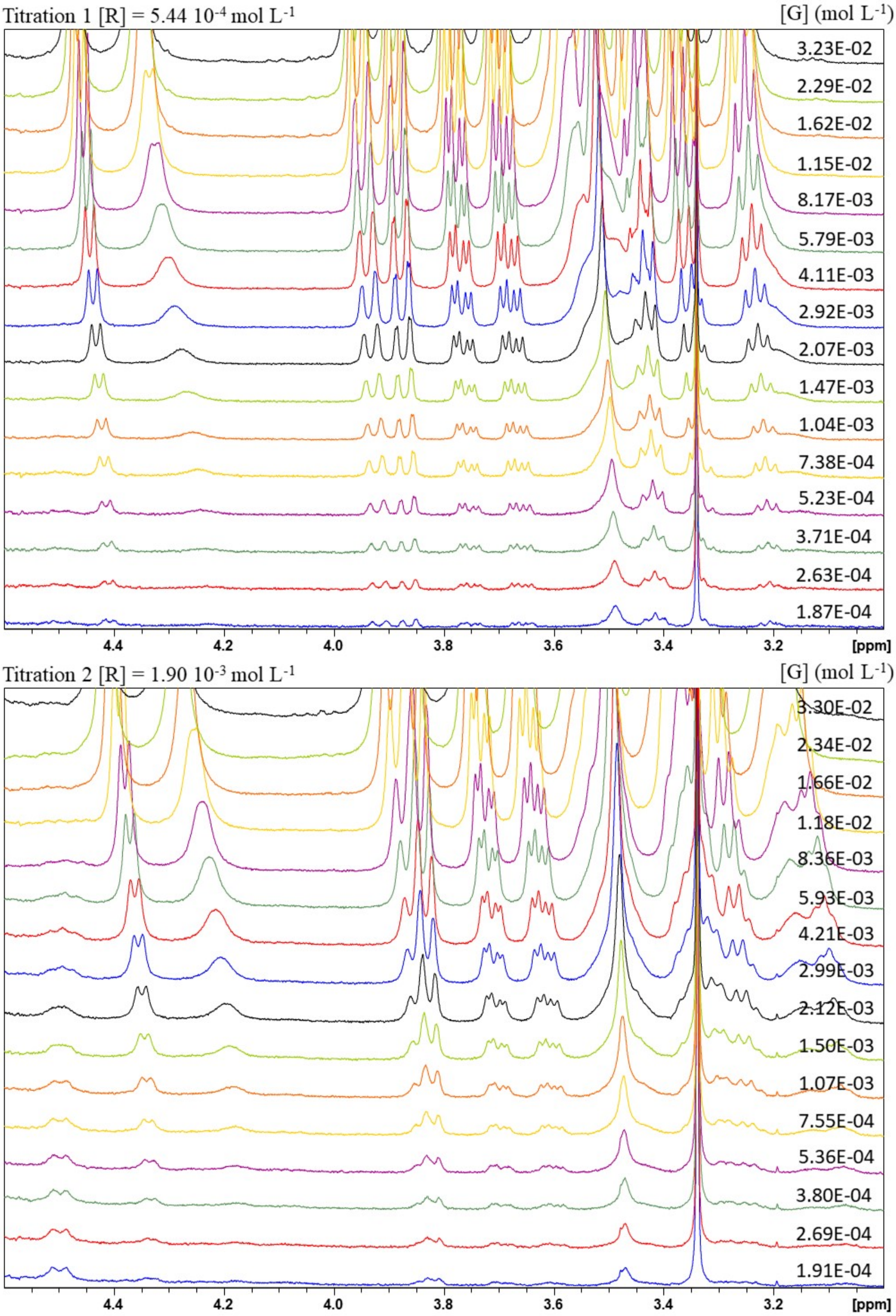

Figure S4. ^1H NMR spectroscopic titrations (500 MHz, D_2O, pD 7.4, 298 K) of receptor **1** (R) with incremental concentrations of MeβCeB (G).

Data Table

R = **1**   G = MeβCeB

δ (ppm) *vs*. [G] (mol L^{-1})

Titration 1 [R] = 5.44 10^{-4} mol L^{-1}

[G]	CH'-1 G	CH-1 G	CH-6 G	CH'-6 G	CH-6' G	CH'-6' G	CH3 G
1.87E-04	4.4164	4.2255	3.9302	3.8756	3.7677	3.6756	3.4881
2.63E-04	4.4176	4.2312	3.9308	3.8764	3.7687	3.6767	3.4896
3.71E-04	4.4201	4.2353	3.9321	3.8774	3.7701	3.6788	3.4920
5.23E-04	4.4224	4.2408	3.9338	3.8784	3.7717	3.6802	3.4948
7.38E-04	4.4259	4.2473	3.9361	3.8802	3.7739	3.6829	3.4977
1.04E-03	4.4304	4.2575	3.9389	3.8822	3.7763	3.6860	3.5023
1.47E-03	4.4351	4.2675	3.9418	3.8842	3.7790	3.6893	3.5068
2.07E-03	4.4407	4.2780	3.9456	3.8864	3.7824	3.6935	3.5123
2.92E-03	4.4462	4.2896	3.9492	3.8889	3.7854	3.6975	3.5175
4.11E-03	4.4524	4.3011	3.9535	3.8918	3.7891	3.7021	3.5234
5.79E-03	4.4581	4.3139	3.9576	3.8945	3.7926	3.7065	3.5288
8.17E-03	4.4642	4.3259	3.9621	3.8973	3.7964	3.7110	3.5341
1.15E-02	4.4696	4.3357	3.9662	3.9001	3.7998	3.7151	3.5388
1.62E-02	4.4746	4.3447	3.9701	3.9024	3.8029	3.7189	3.5429
2.29E-02	4.4789	4.3522	3.9735	3.9045	3.8057	3.7222	3.5462
3.23E-02	4.4831	4.3591	3.9772	3.9066	3.8084	3.7256	3.5494

Titration 2 [R] = 1.90 10^{-3} mol L^{-1}

[G]	CH'-1 G	CH-1 G	CH-6 G	CH'-6 G	CH-6' G	CH'-6' G	CH3 G
1.91E-04	4.3381	4.1777	3.8452	3.8296	3.7108	3.6166	3.4711
2.69E-04	4.3389	4.1783	3.8465	3.8298	3.7117	3.6176	3.4718
3.80E-04	4.3409	4.1790	3.8481	3.8302	3.7125	3.6187	3.4724
5.36E-04	4.3427	4.1805	3.8492	3.8306	3.7136	3.6198	3.4731
7.55E-04	4.3455	4.1834	3.8514	3.8322	3.7147	3.6211	3.4743
1.07E-03	4.3485	4.1854	3.8534	3.8336	3.7167	3.6234	3.4759
1.50E-03	4.3518	4.1908	3.8565	3.8358	3.7191	3.6259	3.4781
2.12E-03	4.3572	4.1962	3.8612	3.8389	3.7225	3.6302	3.4809
2.99E-03	4.3633	4.2062	3.8666	3.8427	3.7267	3.6347	3.4849
4.21E-03	4.3703	4.2157	3.8721	3.8472	3.7311	3.6398	3.4893
5.93E-03	4.3790	4.2276	3.8798	3.8531	3.7372	3.6466	3.4945
8.36E-03	4.3882	4.2406	3.8879	3.8580	3.7435	3.6539	3.5002
1.18E-02	4.3987	4.2542	3.8975	3.8629	3.7503	3.6623	3.5060
1.66E-02	4.4101	4.2690	3.9080	3.8683	3.7581	3.6710	3.5123
2.34E-02	4.4218	4.2842	3.9186	3.8745	3.7661	3.6802	3.5186
3.30E-02	4.4335	4.2989	3.9301	3.8808	3.7743	3.6894	3.5245

Results page

```
 no. of spectra           32
 no. of resonance values  224
 no. of resonant nuclei     7

   sigma =   0.00057332333          RMS weighted residual =   0.00052102849

       stoich            value   relative    log    standard
       coeff                     std devn    beta   deviation

 Beta  0  2 constant 6.9231E+003           3.8403           (   R2   )
 Beta  2  1 refined  8.2635E+004  0.0659    4.9172   0.0286  (   G2R  )
 Beta  1  1 refined  1.8808E+003  0.0452    3.2743   0.0196  (   GR   )
 Beta  1  2 refined  6.5153E+006  0.0514    6.8139   0.0223  (   GR2  )
 Beta  2  2 refined  8.0522E+008  0.1470    8.9059   0.0638  (   G2R2 )

Individual chemical shifts
                   G                       R
 ==========================================================
       +       value    error         value    error
CH'-1   +      4.5078   0.0006
CH-1    +      4.3897   0.0008
CH-6    +      4.0000   0.0007
CH'-6   +      3.9185   0.0005
CH-6'   +      3.8252   0.0006
CH'-6'  +      3.7431   0.0006
CH3     +      3.5620   0.0006
                  0,2                        2,1
 ==========================================================
       +       value    error         value    error
CH'-1   +                            3.3473   0.0206
CH-1    +                            3.0539   0.0263
CH-6    +                            2.8858   0.0195
CH'-6   +                            3.3329   0.0135
CH-6'   +                            3.0258   0.0158
CH'-6'  +                            2.9043   0.0163
CH3     +                            3.0191   0.0148
                  1,1                        1,2
 ==========================================================
       +       value    error         value    error
CH'-1   +      4.2228   0.0122       4.2036   0.0097
CH-1    +      3.5825   0.0336       4.3950   0.0099
CH-6    +      3.8675   0.0068       3.6140   0.0132
CH'-6   +      3.8206   0.0055       3.7115   0.0073
CH-6'   +      3.6775   0.0071       3.5818   0.0083
CH'-6'  +      3.5388   0.0091       3.5103   0.0076
CH3     +      3.1740   0.0166       3.6015   0.0062
                  2,2
 =================================
       +       value    error
CH'-1   +      4.0410   0.0426
CH-1    +      4.5829   0.0468
CH-6    +      3.3057   0.0679
CH'-6   +      3.5649   0.0349
CH-6'   +      3.4060   0.0408
CH'-6'  +      3.3391   0.0381
CH3     +      3.7465   0.0328

 Correlation coefficients*1000
      1    2    3    4
   1
   2  775
   3  152 -388
   4  762  328  525

 Parameters are numbered as follows
    1 beta 2,1
    2 beta 1,1
    3 beta 1,2
    4 beta 2,2
```

Titration Plots

Chemical shifts (δ, ppm) *vs*. concentration of G (mol L^{-1})

experimental (symbols) and calculated (lines) values

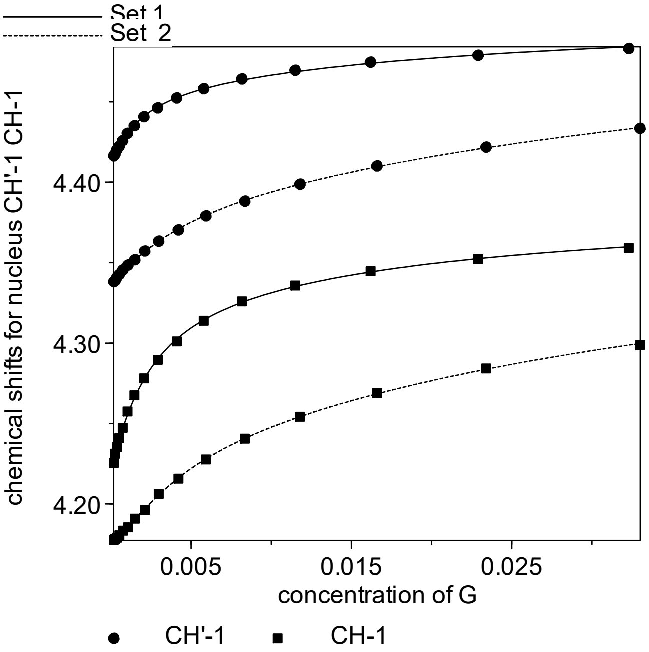

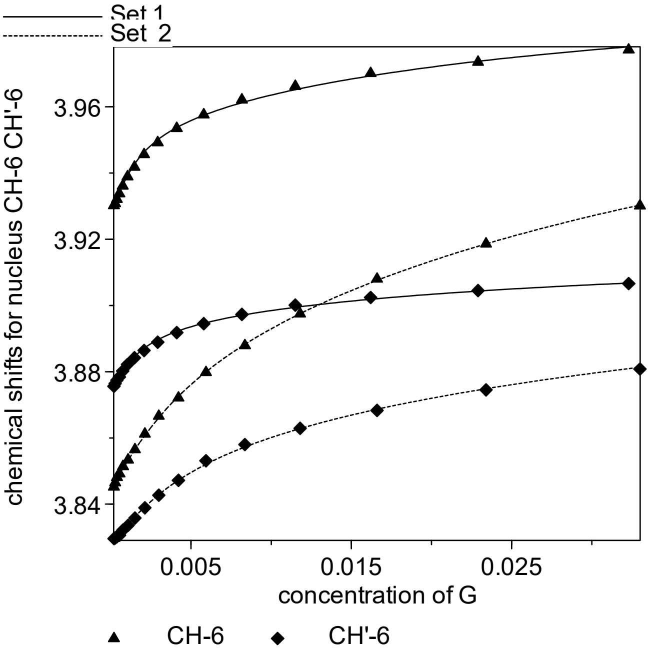

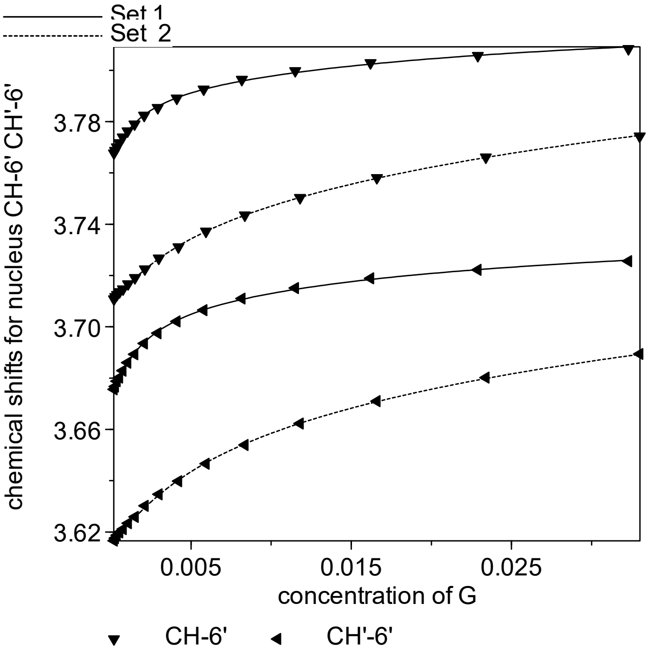

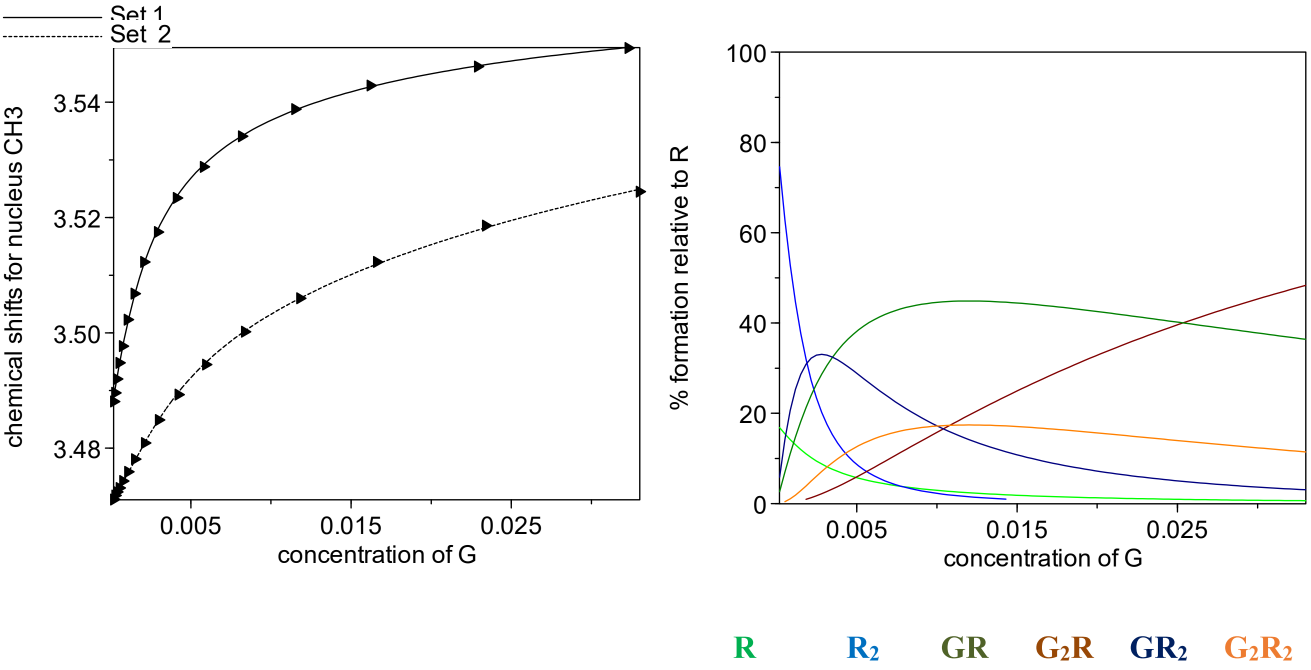
Set 1
Set 2
chemical shifts for nucleus CH3
3.54
3.52
3.50
3.48
0.005
0.015
0.025
concentration of G
% formation relative to R
100
80
60
40
20
0
0.005
0.015
0.025
concentration of G
R
R2
GR
G2R
GR2
G2R2

1 + MeβMal (D_2O, pD 7.4, 298 K, 500 MHz).

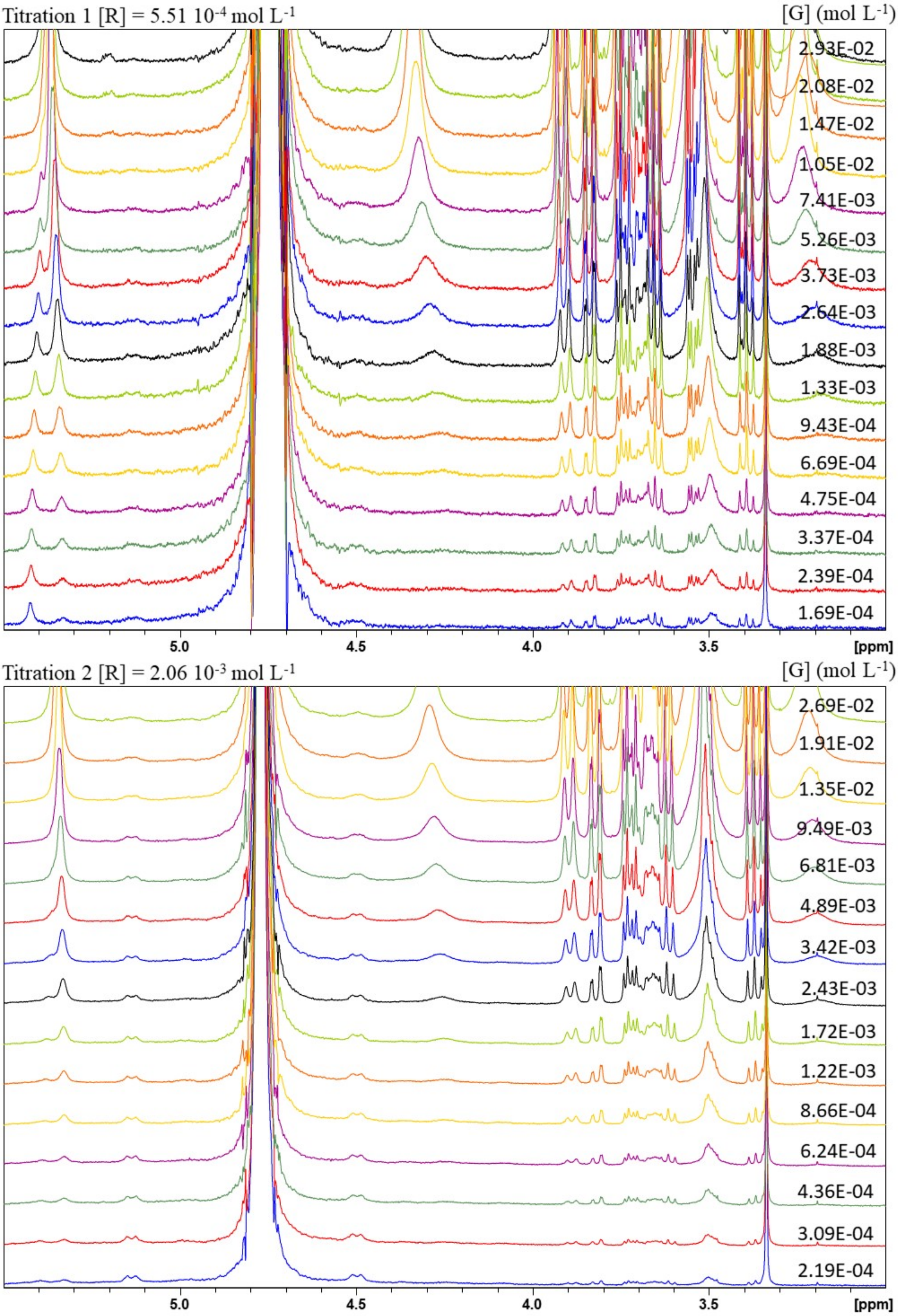

Figure S5. ^{1}H NMR spectroscopic titrations (500 MHz, D_2O, pD 7.4, 298 K) of receptor **1** (R) with incremental concentrations of MeβMal (G).

Data Table

R = **1** G = MeβMal

δ (ppm) *vs*. [G] (mol L^{-1})

Titration 1 [R] = 5.51 10^{-4} mol L^{-1}

[G]	CH'-1 G	CH-1 G	CH-6 G	CH'-6 G	CH'-3 G	CH3 G	CH-2 G
1.69E-04	5.3297	-	3.9148	3.8249	3.6540	3.4917	-
2.39E-04	5.3322	-	3.9159	3.8250	3.6542	3.4949	-
3.37E-04	5.3351	4.2446	3.9171	3.8255	3.6545	3.4962	3.1623
4.75E-04	5.3368	4.2495	3.9179	3.8258	3.6549	3.4989	3.1685
6.69E-04	5.3387	4.2541	3.9188	3.8261	3.6552	3.5012	3.1745
9.43E-04	5.3409	4.2643	3.9200	3.8263	3.6557	3.5049	3.1796
1.33E-03	5.3439	4.2749	3.9216	3.8266	3.6562	3.5088	3.1847
1.88E-03	5.3482	4.2857	3.9230	3.8268	3.6568	3.5138	3.1933
2.64E-03	5.3514	4.2943	3.9251	3.8271	3.6575	3.5184	3.2046
3.73E-03	5.3552	4.3032	3.9270	3.8276	3.6584	3.5236	3.2143
5.26E-03	5.3608	4.3155	3.9299	3.8282	3.6592	3.5313	3.2265
7.41E-03	5.3648	4.3247	3.9319	3.8286	3.6602	3.5366	3.2359
1.05E-02	5.3680	4.3317	3.9336	3.8291	3.6611	3.5394	3.2438
1.47E-02	5.3707	4.3382	3.9350	3.8296	3.6622	3.5418	3.2508
2.08E-02	5.3729	4.3445	3.9364	3.8302	3.6632	3.5444	3.2556
2.93E-02	5.3748	4.3493	3.9375	3.8309	3.6647	3.5469	3.2591

Titration 2 [R] = 2.06 10^{-3} mol L^{-1}

[G]	CH'-1 G	CH-1 G	CH-6 G	CH'-6 G	CH'-3 G	CH3 G	CH-2 G
2.19E-04	5.3268	4.2472	3.9004	3.8064	3.6169	3.5037	-
3.09E-04	5.3274	4.2482	3.9012	3.8068	3.6171	3.5043	-
4.36E-04	5.3281	4.2492	3.9020	3.8071	3.6174	3.5048	-
6.24E-04	5.3286	4.2509	3.9026	3.8073	3.6177	3.5053	3.1828
8.66E-04	5.3291	4.2522	3.9032	3.8075	3.6183	3.5056	3.1834
1.22E-03	5.3305	4.2539	3.9042	3.8079	3.6187	3.5064	3.1849
1.72E-03	5.3310	4.2554	3.9048	3.8082	3.6195	3.5072	3.1867
2.43E-03	5.3325	4.2598	3.9055	3.8086	3.6205	3.5080	3.1886
3.42E-03	5.3339	4.2653	3.9063	3.8091	3.6215	3.5093	3.1916
4.89E-03	5.3364	4.2691	3.9080	3.8097	3.6230	3.5112	3.1974
6.81E-03	5.3393	4.2747	3.9095	3.8107	3.6249	3.5137	3.2028
9.49E-03	5.3420	4.2797	3.9112	3.8117	3.6269	3.5161	3.2082
1.35E-02	5.3449	4.2865	3.9131	3.8127	3.6294	3.5193	3.2136
1.91E-02	5.3477	4.2933	3.9151	3.8142	3.6324	3.5227	3.2202
2.69E-02	5.3510	4.3010	3.9172	3.8158	3.6364	3.5270	3.2256

Results page

```
 no. of spectra            31
 no. of resonance values  210
 no. of resonant nuclei     7

   sigma =   0.00083497849          RMS weighted residual =   0.00075346606

       stoich            value   relative    log    standard
       coeff                     std devn    beta   deviation

 Beta  0  2 constant 6.9231E+003           3.8403           (   R2   )
 Beta  1  1 refined  1.9372E+003  0.1056    3.2872   0.0459  (   GR   )
 Beta  2  1 refined  5.8809E+004  0.1241    4.7694   0.0539  (   G2R  )
 Beta  1  2 refined  6.6479E+006  0.0851    6.8227   0.0370  (   GR2  )
 Beta  2  2 refined  1.6284E+009  0.2169    9.2118   0.0942  (   G2R2 )

 Individual chemical shifts
                   G                        R
 ==========================================================
        +       value    error         value    error
CH'-1   +      5.3883   0.0012
CH-1    +      4.3786   0.0021
CH-6    +      3.9485   0.0011
CH'-6   +      3.8386   0.0009
CH'-3   +      3.6789   0.0014
CH3     +      3.5604   0.0011
CH-2    +      3.2783   0.0016
                 0,2                       1,1
 ==========================================================
        +       value    error         value    error
CH'-1   +                              5.0725   0.0325
CH-1    +                              3.5523   0.0835
CH-6    +                              3.8024   0.0166
CH'-6   +                              3.8227   0.0062
CH'-3   +                              3.6586   0.0075
CH3     +                              3.1328   0.0429
CH-2    +                              2.4938   0.0782
                 2,1                       1,2
 ==========================================================
        +       value    error         value    error
CH'-1   +      4.6967   0.0758         5.4627   0.0103
CH-1    +      2.9497   0.1530         4.6601   0.0289
CH-6    +      3.3377   0.0670         3.9243   0.0053
CH'-6   +      3.3752   0.0524         3.7540   0.0075
CH'-3   +      2.8109   0.0915         3.5066   0.0132
CH3     +      2.9507   0.0690         3.7434   0.0180
CH-2    +      2.4045   0.0983         3.6365   0.0324
                 2,2
 =================================
        +       value    error
CH'-1   +      5.5079   0.0327
CH-1    +      4.8008   0.0997
CH-6    +      3.9535   0.0117
CH'-6   +      3.7510   0.0172
CH'-3   +      3.4855   0.0336
CH3     +      3.7978   0.0551
CH-2    +      3.7073   0.0972

 Correlation coefficients*1000
      1    2    3    4
   1
   2  421
   3 -529  320
   4 -739   -5  619
 Parameters are numbered as follows
    1 beta 1,1
    2 beta 2,1
    3 beta 1,2
    4 beta 2,2
```

Titration Plots

Chemical shifts (δ, ppm) *vs*. concentration of G (mol L^{-1})

experimental (symbols) and calculated (lines) values

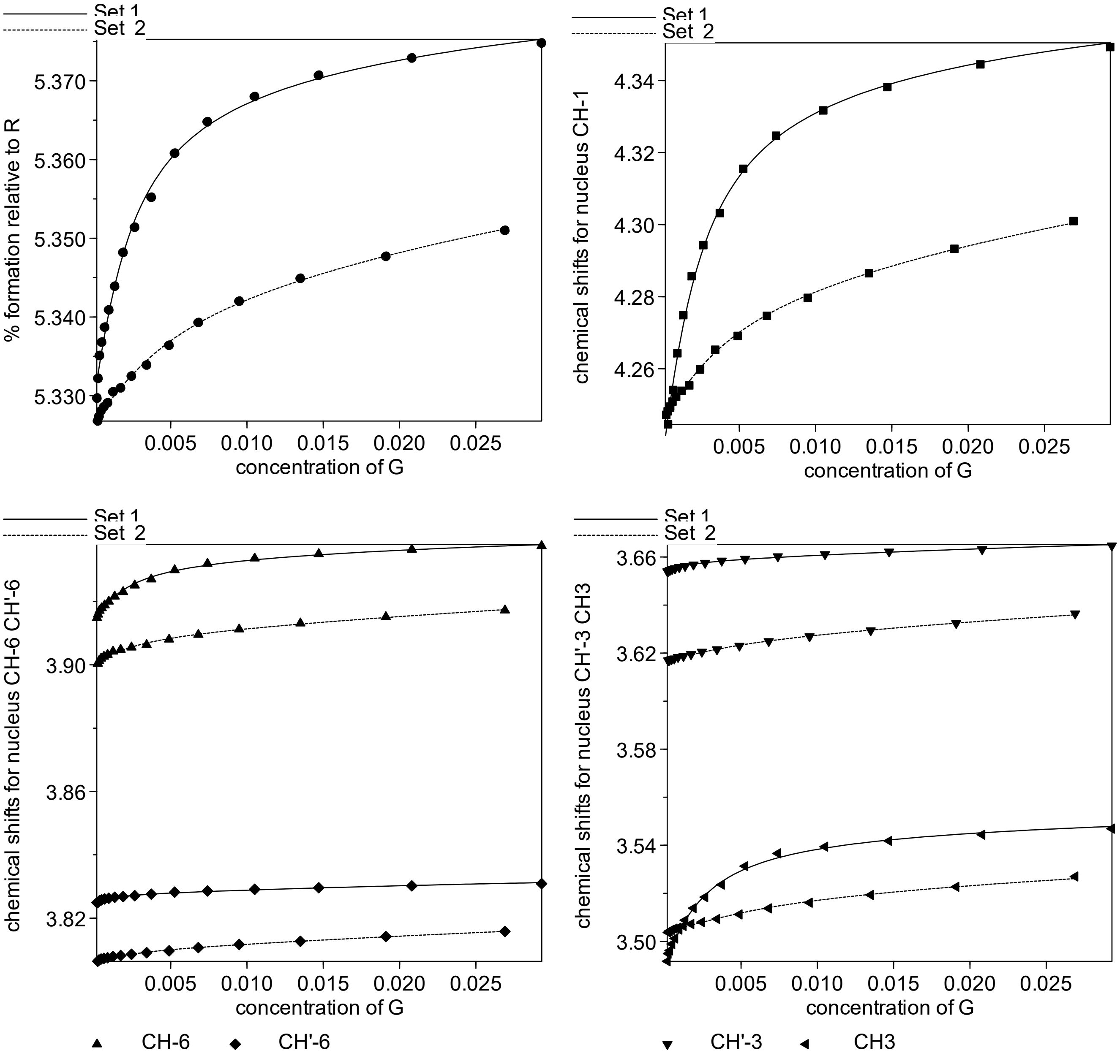

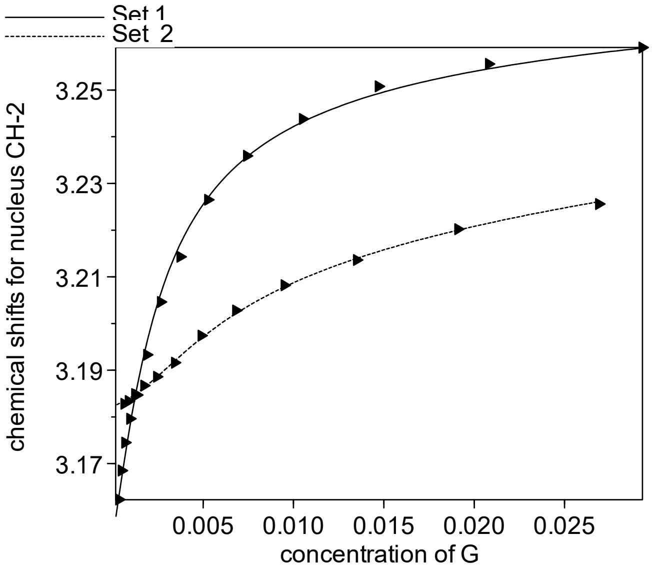

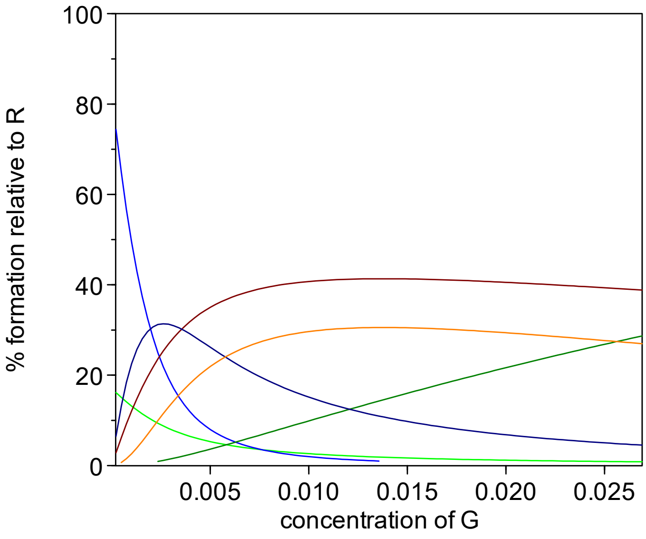

R **R_2** **GR** **G_2R** **GR_2** **G_2R_2**

1 + MeβMal (D_2O, pD 11, 298 K, 500 MHz).

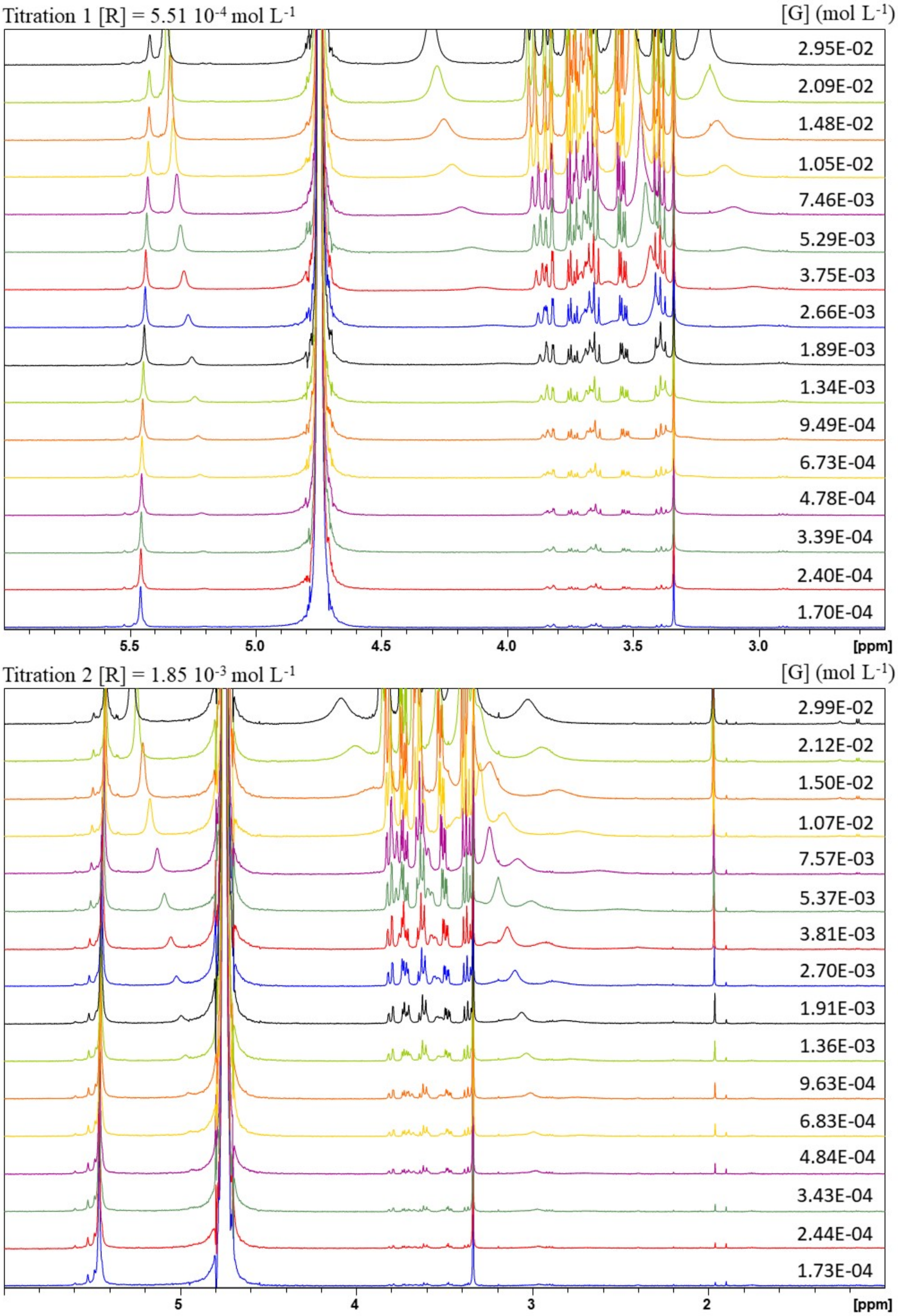

Figure S6. ^{1}H NMR spectroscopic titrations (500 MHz, D_2O, pD 11, 298 K) of receptor **1** (R) with incremental concentrations of MeβMal (G).

Data Table

R = **1** G = MeβMal

δ (ppm) *vs*. [G] (mol L^{-1})

Titration 1 [R] = 5.51 10^{-4} mol L^{-1}

[G]	CH'-1 G	CH-1 G	CH-6 G	CH'-6 G	CH'-3 G	CH'-2 G
0.00E+00	-	-	-	-	-	-
1.70E-04	5.2031	-	-	3.8160	3.6486	3.5144
2.40E-04	5.2058	-	-	3.8165	3.6492	3.5148
3.39E-04	5.2108	-	3.8489	3.8168	3.6497	3.5157
4.78E-04	5.2159	-	3.8512	3.8173	3.6504	3.5167
6.73E-04	5.2221	-	3.8551	3.8177	3.6514	3.5182
9.49E-04	5.2317	3.9635	3.8599	3.8186	3.6523	3.5200
1.34E-03	5.2414	3.9835	3.8658	3.8194	3.6537	3.5221
1.89E-03	5.2556	4.0133	3.8717	3.8203	3.6554	3.5244
2.66E-03	5.2701	4.0631	3.8793	3.8214	3.6573	3.5272
3.75E-03	5.2853	4.1063	3.8869	3.8228	3.6593	3.5303
5.29E-03	5.3001	4.1458	3.8945	3.8239	3.6610	3.5331
7.46E-03	5.3150	4.1842	3.9021	3.8252	3.6628	3.5362
1.05E-02	5.3280	4.2204	3.9090	3.8265	3.6646	3.5388
1.48E-02	5.3403	4.2524	3.9156	3.8275	3.6662	3.5414
2.09E-02	5.3504	4.2805	3.9208	3.8285	3.6675	3.5436
2.95E-02	5.3588	4.3032	3.9256	3.8293	3.6686	3.5454

[G]	CH3 G	CH-2 G	CH-C R	CH-A R	CH-B R	CH-D R
0.00E+00	-	-	8.4368	8.1202	-	7.5320
1.70E-04	-	-	8.4344	8.1203	7.6826	7.5306
2.40E-04	-	-	8.4337	8.1193	7.6803	7.5299
3.39E-04	-	-	8.4332	8.1179	7.6778	7.5294
4.78E-04	-	-	8.4323	8.1163	7.6747	7.5288
6.73E-04	3.3462	2.8245	8.4314	8.1143	7.6708	7.5279
9.49E-04	3.3643	2.8542	8.4299	8.1119	7.6658	7.5267
1.34E-03	3.3809	2.8985	8.4287	8.1087	7.6598	7.5254
1.89E-03	3.3985	2.9347	8.4267	8.1053	7.6524	7.5238
2.66E-03	3.4116	2.9808	8.4249	8.1018	7.6444	7.5223
3.75E-03	3.4330	3.0183	8.4235	8.0979	7.6367	7.5206
5.29E-03	3.4524	3.0604	8.4219	8.0944	7.6293	7.5192
7.46E-03	3.4720	3.1003	8.4203	8.0907	7.6227	7.5178
1.05E-02	3.4891	3.1366	8.4189	8.0877	7.6162	7.5169
1.48E-02	3.5042	3.1686	8.4181	8.0853	7.6112	7.5168
2.09E-02	3.5176	3.1963	8.4173	8.0834	7.6062	7.5167
2.95E-02	3.5285	3.2175	8.4164	8.0820	7.6029	7.5181

Titration 2 [R] = 1.85 10^{-3} mol L^{-1}

[G]	CH'-1 G	CH-1 G	CH-6 G	CH'-6 G	CH'-3 G	CH'-2 G
0.00E+00	-	-	-	-	-	-
1.73E-04	-	-	3.6911	3.7899	3.6185	3.4580
2.44E-04	-	-	3.6945	-	3.6186	3.4587
3.43E-04	-	-	-	3.7903	3.6193	3.4599
4.84E-04	4.9331	-	3.7008	3.7907	3.6201	3.4612
6.83E-04	4.9438	-	3.7049	3.7912	3.6215	3.4627
9.63E-04	4.9570	-	3.7098	3.7922	3.6229	3.4649
1.36E-03	4.9733	-	-	3.7932	3.6249	3.4678
1.91E-03	4.9937	-	-	3.7949	3.6276	3.4716
2.70E-03	5.0213	-	-	3.7967	3.6308	3.4768
3.81E-03	5.0533	-	3.7572	3.7990	3.6346	3.4826
5.37E-03	5.0922	-	3.7765	3.8021	3.6395	3.4896
7.57E-03	5.1321	-	3.7989	3.8049	3.6444	3.4972
1.07E-02	5.1727	-	3.8145	3.8080	3.6493	3.5048
1.50E-02	5.2125	3.9069	3.8362	3.8111	3.6546	3.5124
2.12E-02	5.2462	3.9986	3.8535	3.8137	3.6589	3.5189
2.99E-02	5.2748	4.0878	3.8684	3.8160	3.6622	3.5249

[G]	CH3 G	CH-2 G	CH-C R	CH-A R	CH-B R	CH-D R
-	-	8.4388	8.1202	7.6968	7.5238	-
2.9623	-	8.4381	8.1192	7.6927	7.5232	2.9623
2.9673	-	8.4376	8.1186	7.6914	7.5229	2.9673
2.9728	-	8.4372	8.1178	7.6897	7.5226	2.9728
2.9814	-	8.4366	8.1166	7.6873	7.5220	2.9814
2.9962	-	8.4358	8.1151	7.6845	7.5213	2.9962
3.0125	-	8.4346	8.1131	7.6802	7.5203	3.0125
3.0353	-	8.4334	8.1106	7.6754	7.5191	3.0353
3.0636	2.2048	8.4317	8.1074	7.6688	7.5177	3.0636
3.0991	2.3039	8.4298	8.1041	7.6615	7.5161	3.0991
3.1428	2.4041	8.4276	8.0999	7.6532	7.5141	3.1428
3.1940	2.5139	8.4259	8.0956	7.6443	7.5123	3.1940
3.2464	2.6241	8.4238	8.0913	7.6355	7.5105	3.2464
3.3011	2.7458	8.4213	8.0876	7.6273	7.5091	3.3011
3.3512	2.8614	8.4199	8.0842	7.6202	7.5081	3.3512
3.3988	2.9499	8.4191	8.0816	7.6145	7.5080	3.3988
3.4337	3.0286	8.4188	8.0798	7.6105	7.5083	3.4337

Results page

```
no. of spectra            34
no. of resonance values  348
no. of resonant nuclei    12

  sigma =   0.00146502824          RMS weighted residual =   0.00131412110
```

stoich coeff				value	relative std devn	log beta	standard deviation	
Beta	0	2	constant	8.9084E+003		3.9498		(R2)
Beta	2	1	refined	9.0798E+003	0.8722	3.9581	0.3788	(G2R)
Beta	1	1	refined	6.5012E+002	0.1017	2.8130	0.0442	(GR)
Beta	1	2	refined	7.5419E+006	0.0851	6.8775	0.0370	(GR2)
Beta	2	2	refined	6.3443E+008	0.1676	8.8024	0.0728	(G2R2)

Individual chemical shifts

		G		R	
	+	value	error	value	error
CH'-1	+	5.3908	0.0035		
CH-1	+	4.3951	0.0074		
CH-6	+	3.9551	0.0026		
CH'-6	+	3.8373	0.0019		
CH'-3	+	3.6693	0.0019		
CH'-2	+	3.5553	0.0019		
CH3	+	3.5598	0.0042		
CH-2	+	3.2794	0.0076		
CH-C	+			8.4123	0.0062
CH-A	+			8.1310	0.0062
CH-B	+			7.6202	0.0067
CH-D	+			7.5865	0.0063

		0,2		2,1	
	+	value	error	value	error
CH'-1	+			3.4381	0.5478
CH-1	+			-0.7745	1.2849
CH-6	+			1.1418	1.6634
CH'-6	+			2.9332	0.6466
CH'-3	+			3.8785	0.3460
CH'-2	+			2.6531	0.5380
CH3	+			2.4954	0.6445
CH-2	+			1.3365	1.3485
CH-C	+	8.4442	0.0018	8.4276	0.0204
CH-A	+	8.1186	0.0018	8.0955	0.0264
CH-B	+	7.7109	0.0019	7.6310	0.0367
CH-D	+	7.5120	0.0018	7.5411	0.0335

		1,1		1,2	
	+	value	error	value	error
CH'-1	+	5.1468	0.0613	4.1173	0.0632
CH-1	+	3.2900	0.2378	1.1400	0.1865
CH-6	+	3.6697	0.0528	3.2897	0.0351
CH'-6	+	3.7560	0.0409	3.7259	0.0114
CH'-3	+	3.6359	0.0401	3.5337	0.0115
CH'-2	+	3.4431	0.0412	3.3117	0.0152
CH3	+	3.2921	0.0702	1.9282	0.0826
CH-2	+	2.9469	0.1411	-0.4437	0.1868
CH-C	+	8.4075	0.0059	8.4318	0.0026
CH-A	+	8.0830	0.0061	8.1019	0.0027
CH-B	+	7.5704	0.0105	7.6715	0.0029
CH-D	+	7.5361	0.0063	7.5047	0.0026

		2,2	
	+	value	error
CH'-1	+	2.4223	0.2188
CH-1	+	-3.4904	0.5299
CH-6	+	2.4048	0.1251
CH'-6	+	3.5691	0.0390
CH'-3	+	3.3451	0.0389
CH'-2	+	2.9732	0.0544

```
CH3     +      -0.1605   0.2732
CH-2    +      -4.3302   0.5151
CH-C    +       8.4174   0.0044
CH-A    +       8.0614   0.0047
CH-B    +       7.6033   0.0053
CH-D    +       7.4799   0.0055

 Correlation coefficients*1000

      1    2    3    4
   1
   2  724
   3  -72 -553
   4   90 -450  957

 Parameters are numbered as follows
    1 beta 2,1
    2 beta 1,1
    3 beta 1,2
    4 beta 2,2
```

Titration Plots

Chemical shifts (δ, ppm) *vs*. concentration of G (mol L^{-1})

experimental (symbols) and calculated (lines) values

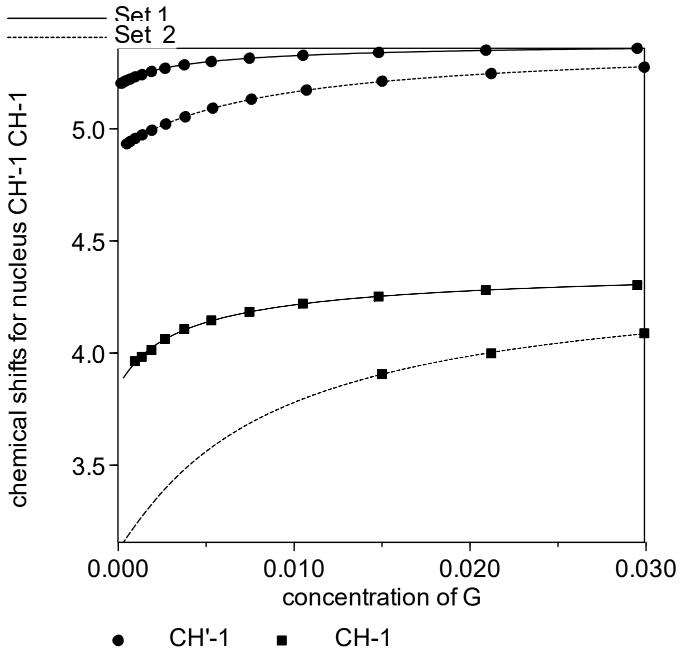

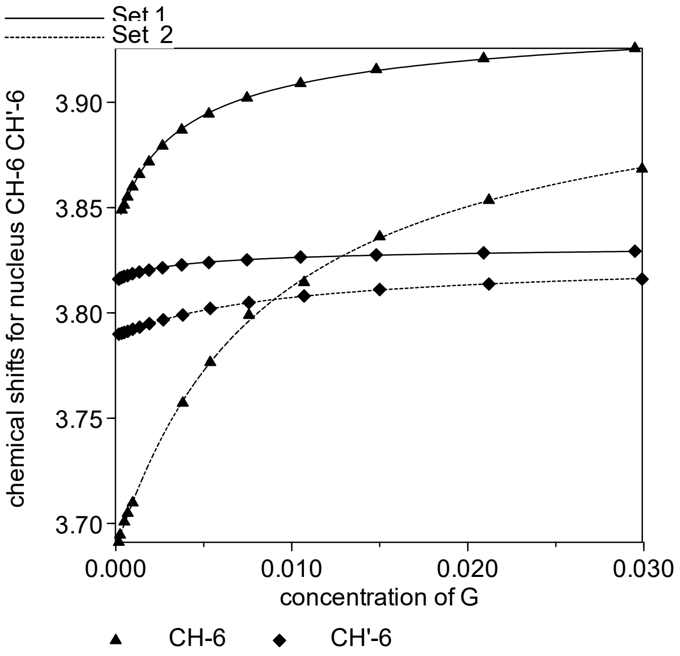

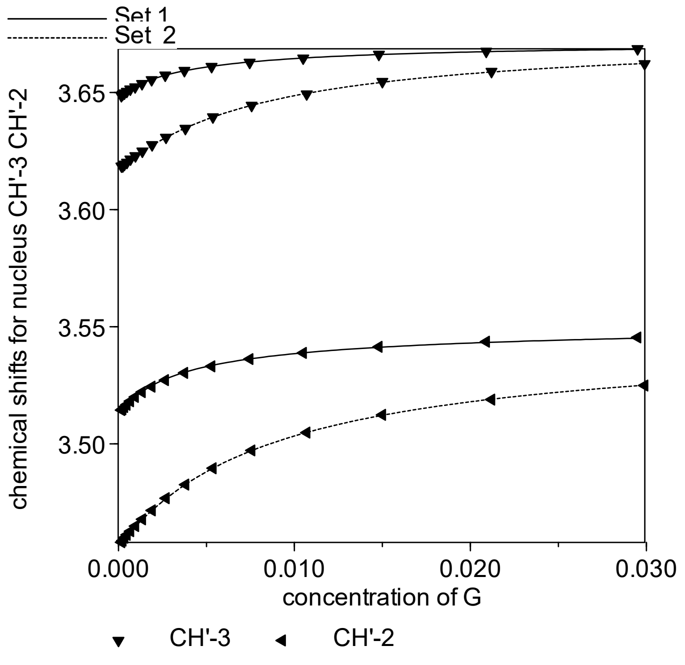

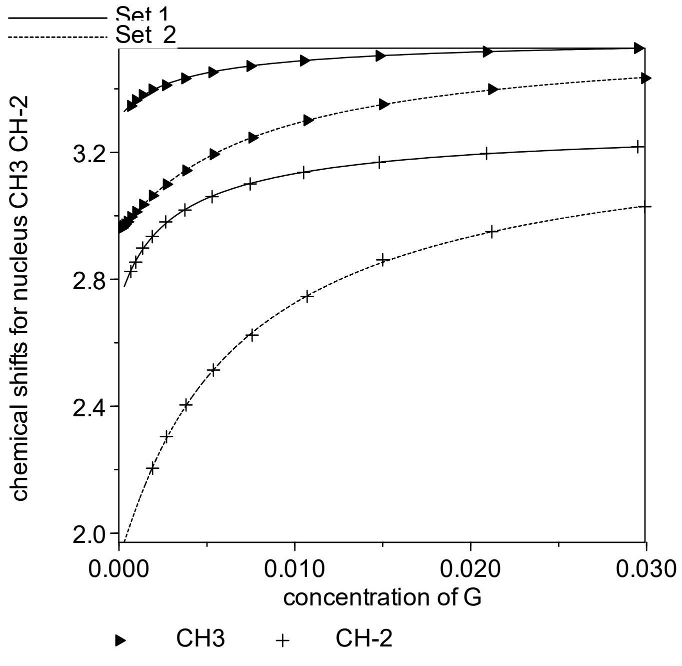

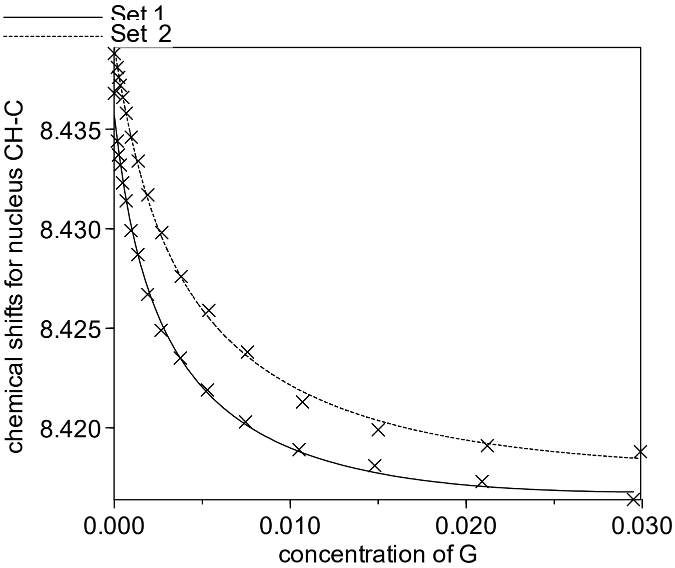
Set 1
Set 2
chemical shifts for nucleus CH-C
8.435
8.430
8.425
8.420
0.000
0.010
0.020
0.030
concentration of G

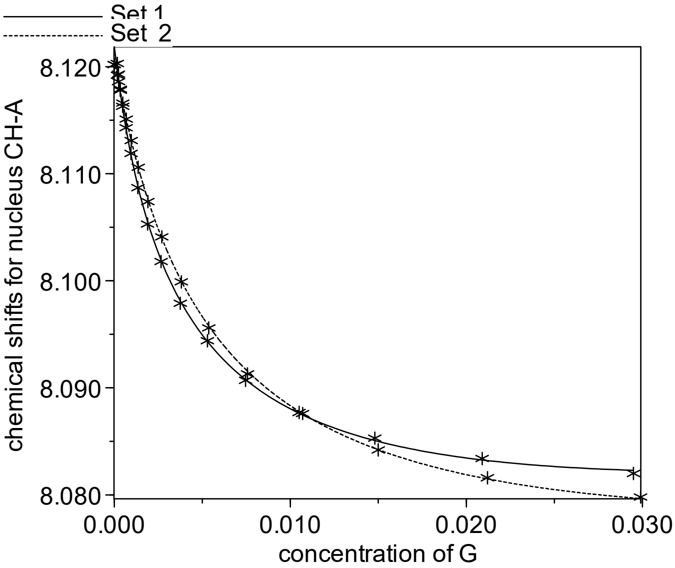
Set 1
Set 2
chemical shifts for nucleus CH-A
8.120
8.110
8.100
8.090
8.080
0.000
0.010
0.020
0.030
concentration of G

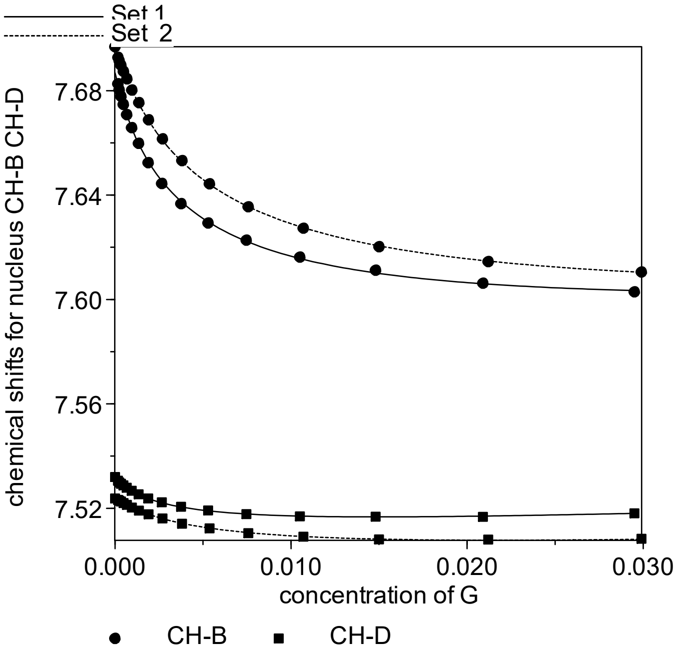
Set 1
Set 2
chemical shifts for nucleus CH-B CH-D
7.68
7.64
7.60
7.56
7.52
0.000
0.010
0.020
0.030
concentration of G
CH-B
CH-D

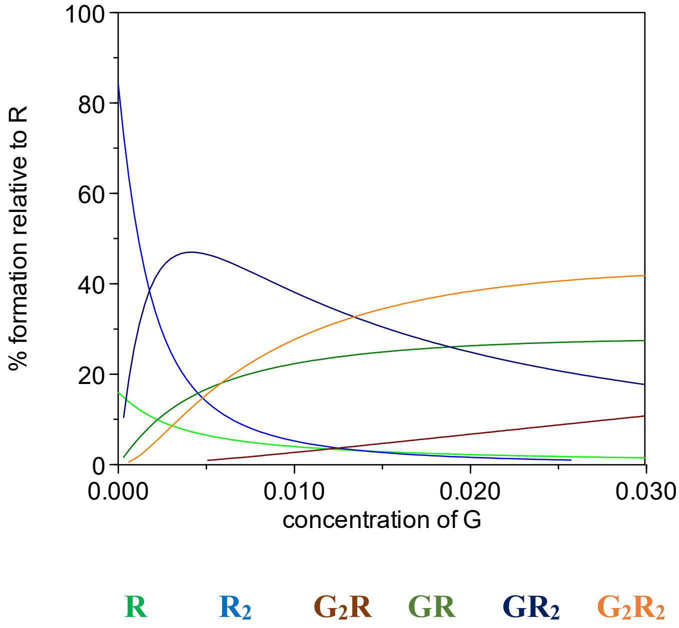
% formation relative to R
100
80
60
40
20
0
0.000
0.010
0.020
0.030
concentration of G
R
R2
G2R
GR
GR2
G2R2

1 + MeβLac (D_2O, pD 7.4, 298 K, 500 MHz).

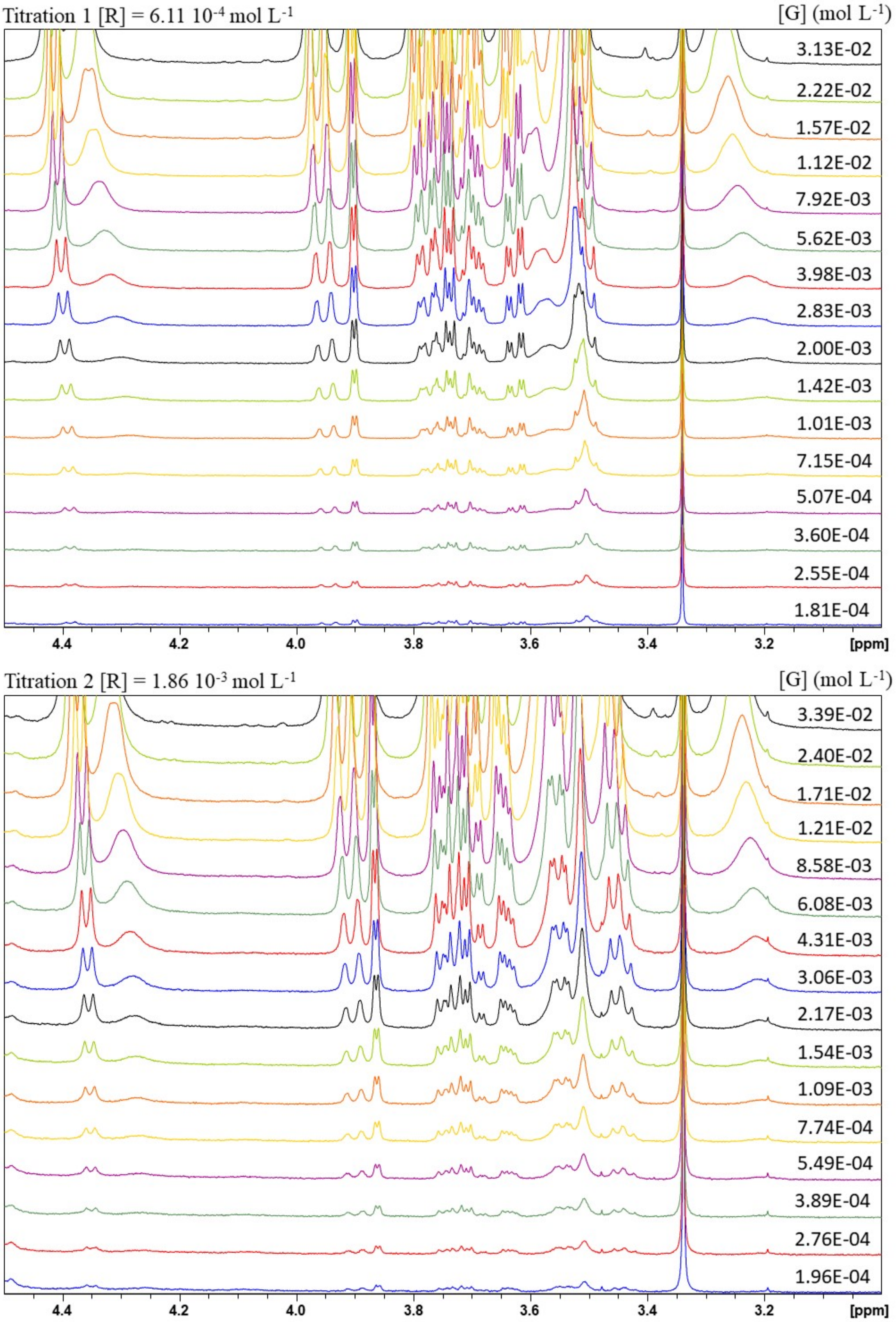

Figure S7. ^{1}H NMR spectroscopic titrations (500 MHz, D_2O, pD 7.4, 298 K) of receptor **1** (R) with incremental concentrations of MeβLac (G).

Data Table

R = **1**   G = MeβLac

δ (ppm) *vs*. [G] (mol L^{-1})

Titration 1 [R] = 6.11 10^{-4} mol L^{-1}

[G]	CH-1 G	CH'-1 G	CH'-6 G	CH-4 G	CH3 G	CH'-2 G
1.81E-04	4.3945	-	3.9571	3.9034	-	-
2.55E-04	4.3952	4.2749	3.9579	3.9035	-	3.1855
3.60E-04	4.3957	4.2757	3.9584	3.9036	-	3.1863
5.07E-04	4.3962	4.2767	3.9592	3.9038	-	3.1874
7.15E-04	4.3977	-	3.9599	3.9039	-	3.1888
1.01E-03	4.3994	-	3.9607	3.9042	-	3.1921
1.42E-03	4.4018	4.2922	3.9623	3.9046	3.5124	-
2.00E-03	4.4042	4.3002	3.9640	3.9051	3.5173	3.2098
2.83E-03	4.4071	4.3086	3.9656	3.9055	3.5226	3.2165
3.98E-03	4.4105	4.3181	3.9678	3.9061	3.5256	3.2269
5.62E-03	4.4136	4.3286	3.9699	3.9067	3.5310	3.2364
7.92E-03	4.4172	4.3383	3.9724	3.9074	3.5363	3.2463
1.12E-02	4.4205	4.3473	3.9744	3.9082	3.5411	3.2548
1.57E-02	4.4237	4.3554	3.9771	3.9092	3.5452	3.2629
2.22E-02	4.4264	4.3622	3.9793	3.9099	3.5485	3.2691
3.13E-02	4.4290	4.3682	3.9811	3.9109	3.5516	3.2749

Titration 2 [R] = 1.86 10^{-4} mol L^{-1}

[G]	CH-1 G	CH'-1 G	CH'-6 G	CH-4 G	CH3 G	CH'-2 G
1.96E-04	4.3587	-	3.9111	3.8645	3.5081	-
2.76E-04	4.3589	-	3.9115	3.8648	3.5085	-
3.89E-04	4.3592	4.2650	3.9119	3.8651	3.5088	-
5.49E-04	4.3595	4.2685	3.9124	3.8654	3.5091	3.2019
7.74E-04	4.3600	4.2699	3.9130	3.8657	3.5095	3.2023
1.09E-03	4.3609	4.2719	3.9138	3.8660	3.5100	3.2039
1.54E-03	4.3619	4.2751	3.9144	3.8665	3.5108	3.2056
2.17E-03	4.3636	4.2780	3.9158	3.8672	3.5121	3.2085
3.06E-03	4.3652	4.2800	3.9176	3.8681	3.5133	3.2108
4.31E-03	4.3675	4.2852	3.9196	3.8694	3.5151	3.2144
6.08E-03	4.3706	4.2901	3.9223	3.8711	3.5172	3.2187
8.58E-03	4.3743	4.2969	3.9256	3.8729	3.5205	3.2247
1.21E-02	4.3785	4.3036	3.9294	3.8749	3.5239	3.2305
1.71E-02	4.3834	4.3127	3.9341	3.8775	3.5281	3.2377
2.40E-02	4.3889	4.3217	3.9395	3.8806	3.5322	3.2452
3.39E-02	4.3948	4.3308	3.9451	3.8841	3.5362	3.2528

Results page

```
no. of spectra             32
no. of resonance values  173
no. of resonant nuclei     6

  sigma =   0.00045085220          RMS weighted residual =   0.00040412763

      stoich            value   relative    log    standard
      coeff                     std devn    beta   deviation

Beta  0  2 constant 6.9231E+003            3.8403            (   R2   )
Beta  1  1 refined  1.5380E+003  0.0287    3.1870   0.0125  (   GR   )
Beta  2  1 refined  2.6093E+004  0.0524    4.4165   0.0228  (   G2R  )
Beta  1  2 refined  1.6783E+006  0.0883    6.2249   0.0383  (   GR2  )
Beta  2  2 refined  1.0965E+008  0.3043    8.0400   0.1322  (   G2R2 )

Individual chemical shifts

                   G                        R
 =========================================================
       +        value    error         value    error
CH'-1   +       4.4496   0.0007
CH-1    +       4.3936   0.0008
CH-6    +       4.0022   0.0007
CH'-4   +       3.9261   0.0006
CH3     +       3.5620   0.0005
CH-2    +       3.2913   0.0007
                  0,2                      1,1
 =========================================================
       +        value    error         value    error
CH'-1   +                               4.2138   0.0084
CH-1    +                               3.5984   0.0237
CH-6    +                               3.8750   0.0060
CH'-4   +                               3.9186   0.0040
CH3     +                               3.1514   0.0132
CH-2    +                               2.4855   0.0237
                  2,1                      1,2
 =========================================================
       +        value    error         value    error
CH'-1   +       3.0887   0.0478         4.2617   0.0163
CH-1    +       3.0443   0.0499         5.4141   0.0908
CH-6    +       2.5299   0.0511         3.5179   0.0393
CH'-4   +       2.8070   0.0402         3.3825   0.0451
CH3     +       3.0565   0.0248         4.1991   0.0558
CH-2    +       2.6018   0.0322         4.6695   0.1197
                  2,2
 =================================
       +        value    error
CH'-1   +       4.4177   0.0499
CH-1    +       6.8388   0.7318
CH-6    +       3.4126   0.1684
CH'-4   +       3.1621   0.2201
CH3     +       4.8938   0.3952
CH-2    +       6.1712   0.8524

Correlation coefficients*1000

      1    2    3    4
  1
  2  280
  3 -647  208
  4 -544  236  498

Parameters are numbered as follows
   1 beta 1,1
   2 beta 2,1
   3 beta 1,2
   4 beta 2,2
```

Titration Plots

Chemical shifts (δ, ppm) *vs*. concentration of G (mol L^{-1})

experimental (symbols) and calculated (lines) values

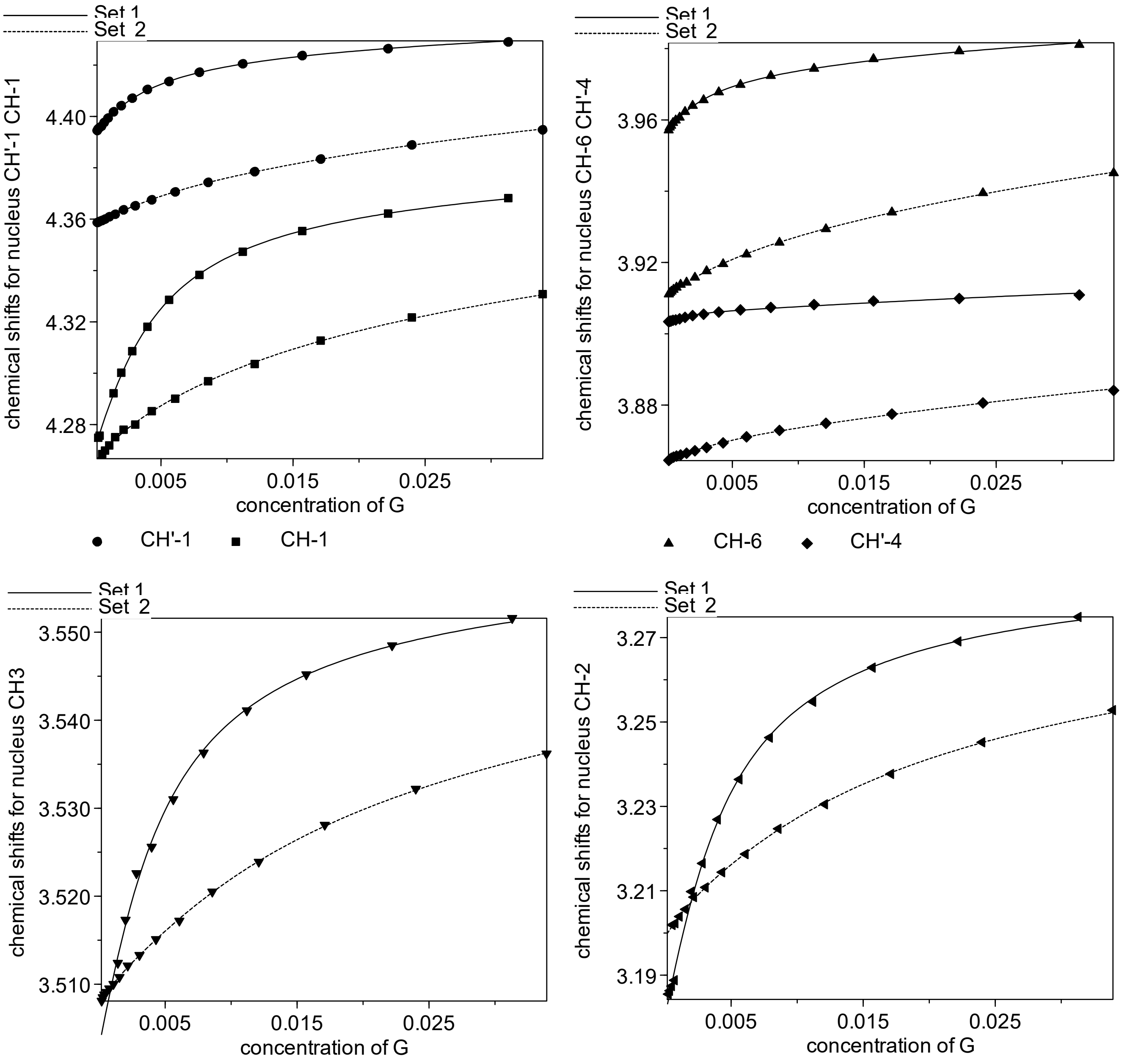

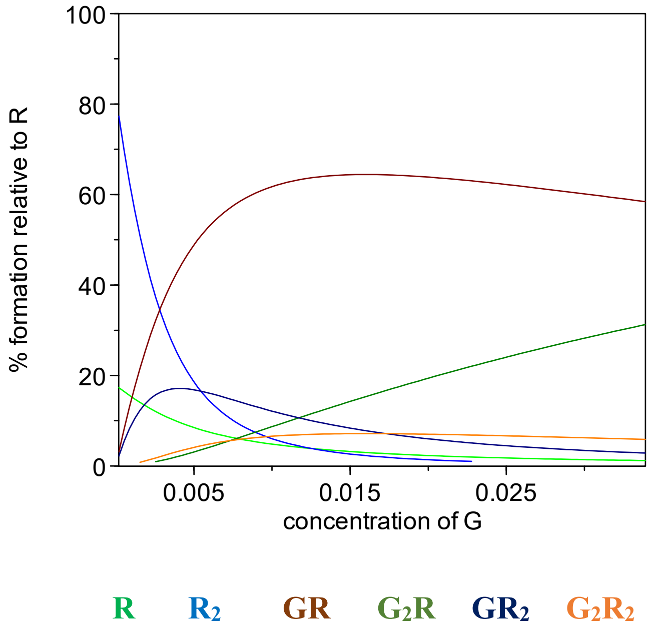
100
80
60
40
20
0
% formation relative to R
0.005
0.015
0.025
concentration of G
R
R_2
GR
G_2R
GR_2
G_2R_2

1 + MeβGlcNAc2 (D_2O, pD 7.4, 298 K, 500 MHz).

R = **1**   G = MeβGlcNAc2

Titration

[R] = 8.54 10^{-4} mol L^{-}

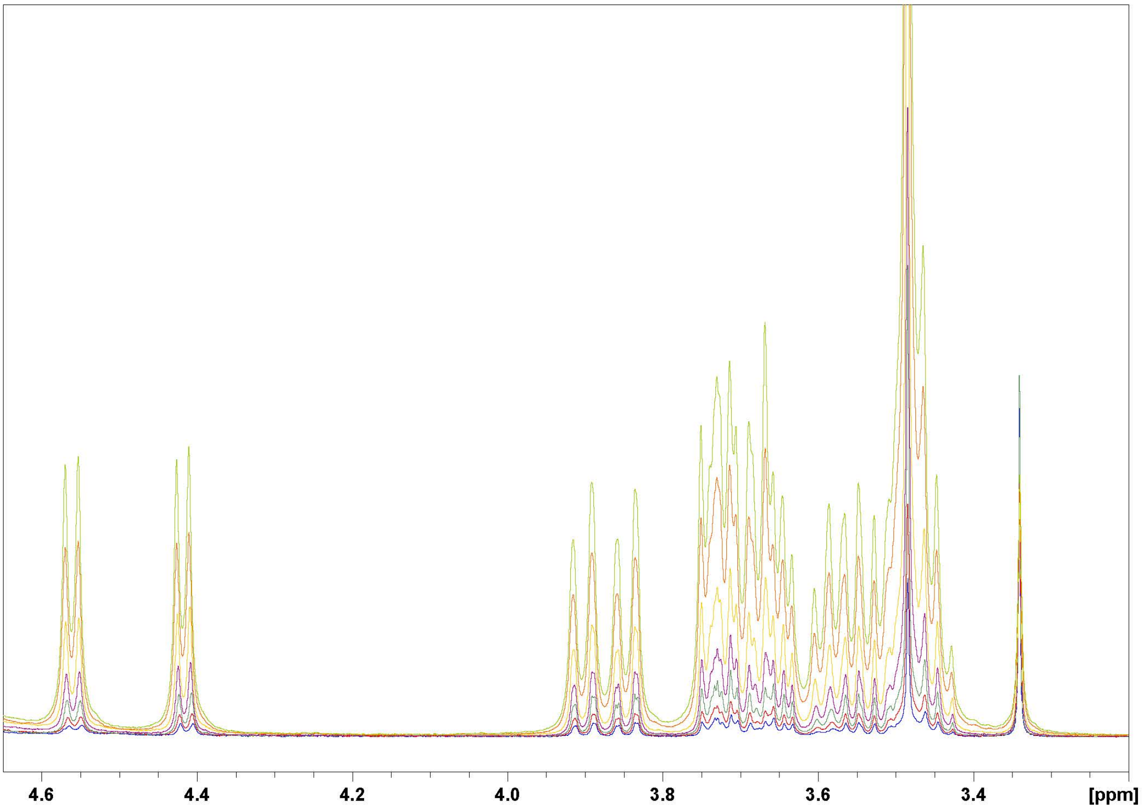

Figure S8. Superposition of ^{1}H NMR spectra registered at incremental concentrations of MeβGlcNAc2 (0.904 mM, 1.64 mM, 2.99 mM, 5.45 mM, 10.8 mM, 21.6 mM, 30.4 mM) in a 0.854 mM solution of **1**. Expansion of the saccharide region.

Table S1. Cumulative formation constants (log β_n)[a] and intrinsic median binding concentration (BC_{50}^0, mM)[b] for receptor **1** to MeβMal (R:G) complexes, measured at 298 K from NMR data in D_2O at pD 11.[c]

R:G	log β_n	BC_{50}^0
1:1	2.81±0.04	1.93±0.40
1:2	3.96±0.38	
2:1	6.88±0.04	
2:2	8.80±0.07	

[a] Formation constants were obtained by nonlinear least-square regression analysis of NMR data. [b] Calculated from the log β values using the "BC50 Calculator" program.[16] [c] Receptor dimerization constant at pD 11 (**1**: log β_{dim} = 3.95±0.11) was set invariant in the nonlinear regression analysis of NMR data.

Structural studies.

Chemical shift difference (CSD) analysis

Table S2. Chemical shifts and chemical shift differences (CSD) [ppm] of the anomeric protons of MeβCeB, MeβMal and MeβLac in free and 1:1 bound state with **1** in D_2O (pD 7.4) at T=298 K[a]

	Nucleus	free	bound	CSD
MeβCeB	H'-1	4.5078	4.2228	0.2850
	H-1	4.3897	3.5825	0.8072
MeβMal	H'-1	5.3883	5.0725	0.3158
	H-1	4.3786	3.5523	0.8263
MeβLac	H'-1	4.4496	4.2138	0.2358
	H-1	4.3936	3.5984	0.7952

[a] Chemical shifts were obtained by nonlinear regression of the experimental data from titration of MeβCeB, MeβMal and MeβLac with **1**.

Table S3. Chemical shifts and chemical shift differences (CSD) [ppm] of the anomeric protons of MeβCeB, MeβMal and MeβLac in free and 1:1 bound state with **2** in D_2O (pD 7.4) at T=298 K[a]

	Nucleus	free	bound	CSD
MeβCeB	H'-1	4.5078	0.7440	3.7638
	H-1	4.3897	0.8405	3.5492
MeβMal	H'-1	5.3883	3.9648	1.4235
	H-1	4.3786	2.3238	2.0548
MeβLac	H'-1	4.4496	3.1512	1.2984
	H-1	4.3936	2.9625	1.4311

[a] Chemical shifts were obtained by nonlinear regression of the experimental data from titration of MeβCeB, MeβMal and MeβLac with **2**.

NMR methods. NMR experiments were performed at 500 MHz in D_2O at pD 11 at 298 K. The experiments on the complex were performed using an equimolar solution of **1** or **2** (R) and MeβCeB (G). In addition to standard 1D ^1H NMR spectra, COSY, TOCSY, HSQC and NOESY experiments (500 ms mixing time) were also acquired to assign the resonances of all the molecular entities and to detect the relevant intramolecular and intermolecular contacts.

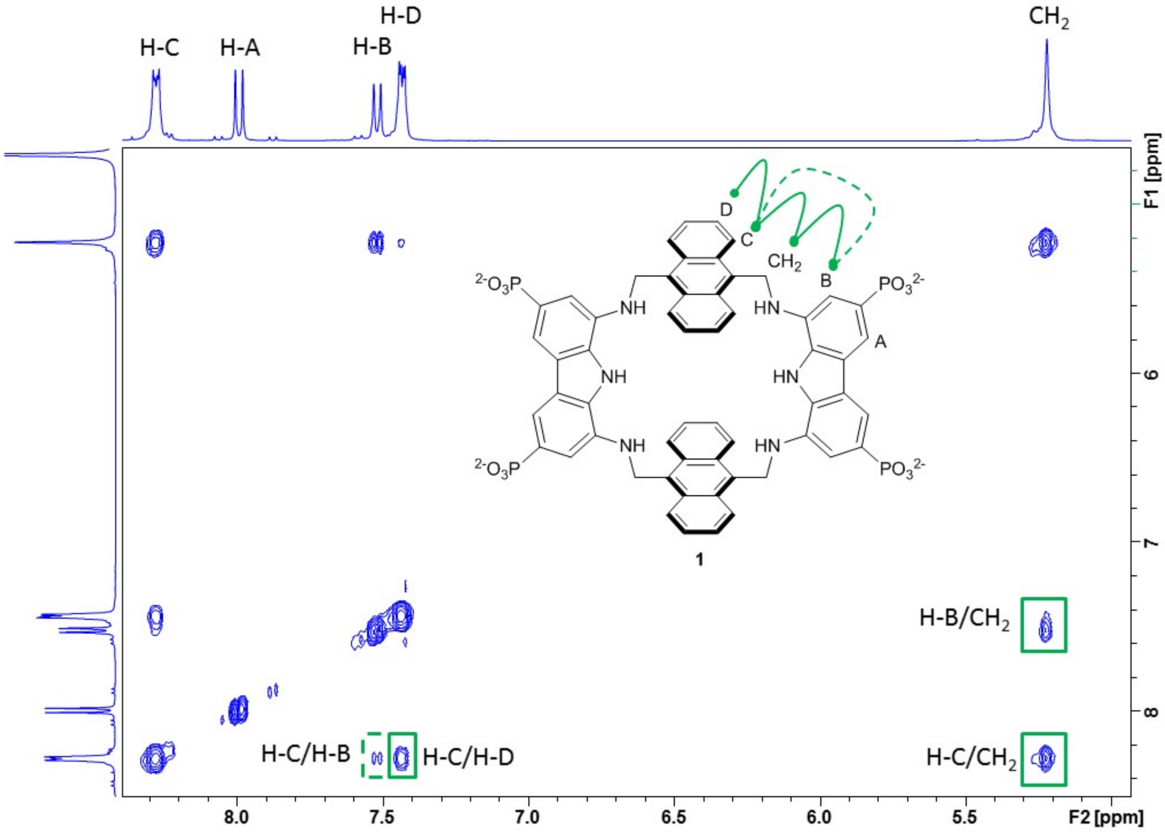

Figure S9. 500 MHz NOESY spectrum of an equimolar mixture of MeβCeB and **1** (30 mM each) in D_2O at 298 K. Intramolecular NOE cross peaks of the receptor **1** are indicated by squares and schematically represented (solid and dashed squares and lines were used to indicate strong and medium NOEs found).

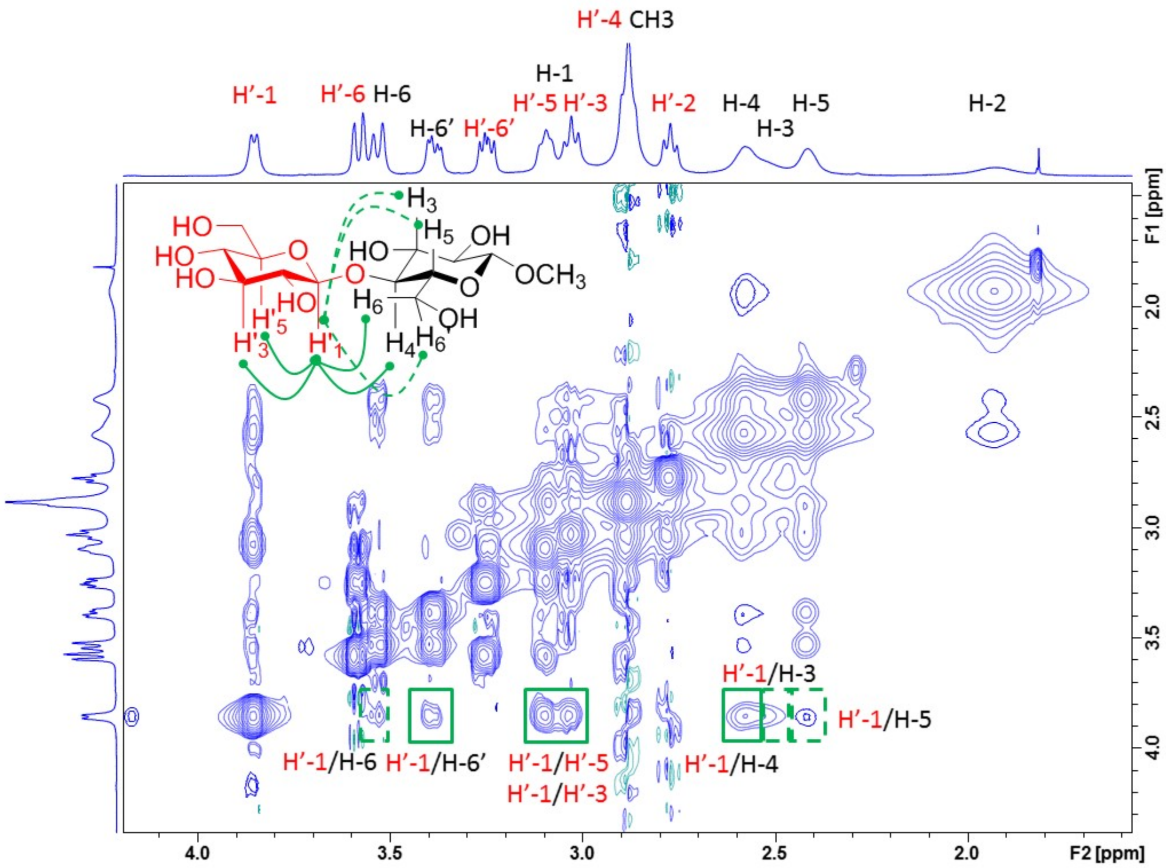

Figure S10. 500 MHz NOESY spectrum of an equimolar mixture of MeβCeB and **1** (30 mM each) in D_2O (pD 11) at 298 K. Intramolecular NOE cross peaks of MeβCeB relating to H'-1 proton are indicated by squares and schematically represented (solid line and dashed lines were used to indicate strong and weak NOEs found).

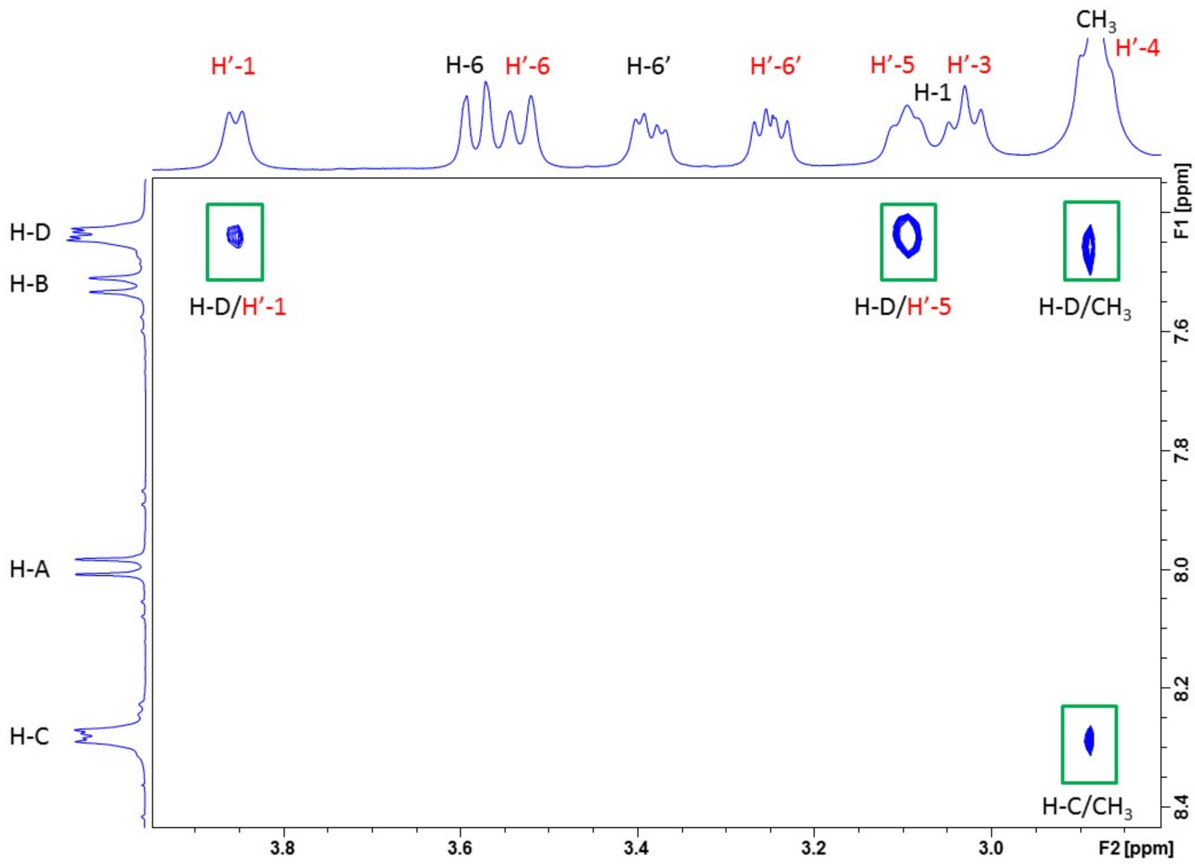

Figure S11. 500 MHz NOESY spectrum of an equimolar mixture of MeβCeB and **1** (30 mM each) in D_2O (pD 11) at 298 K. Strong intermolecular NOE cross peaks are indicated by solid squares.

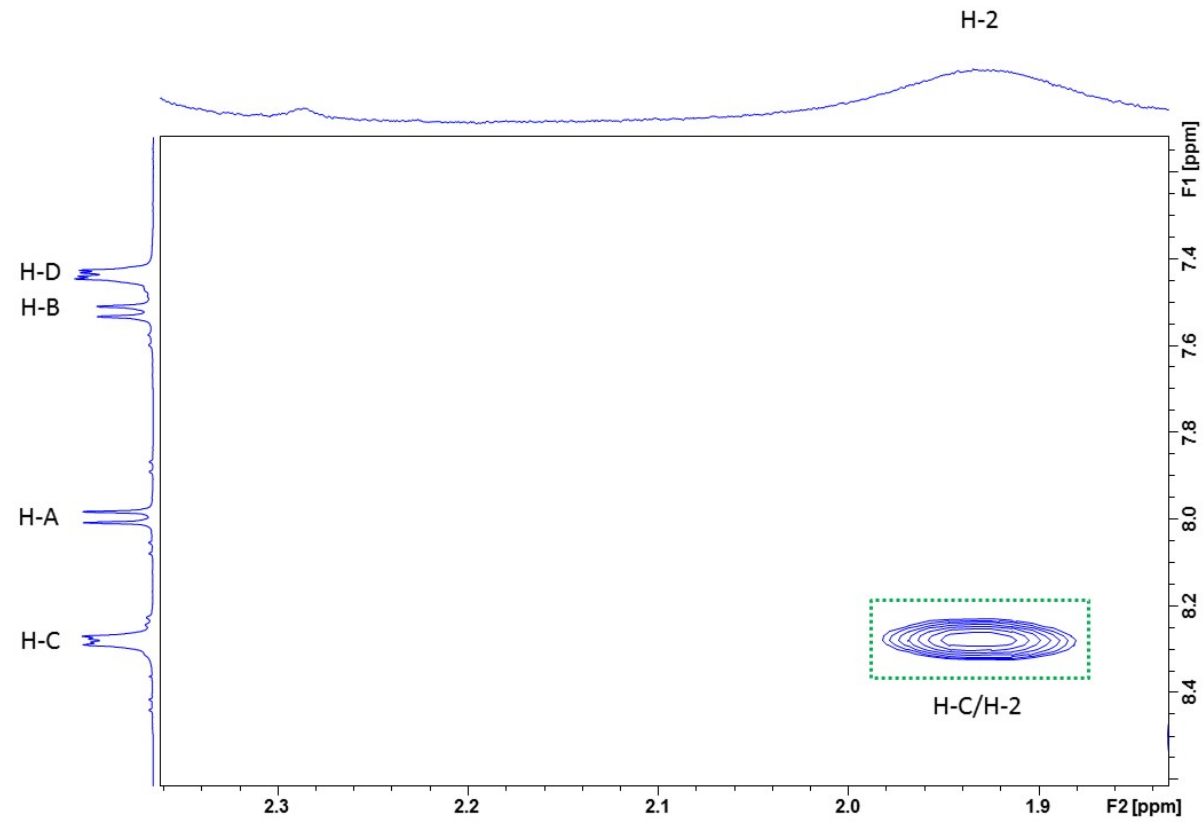

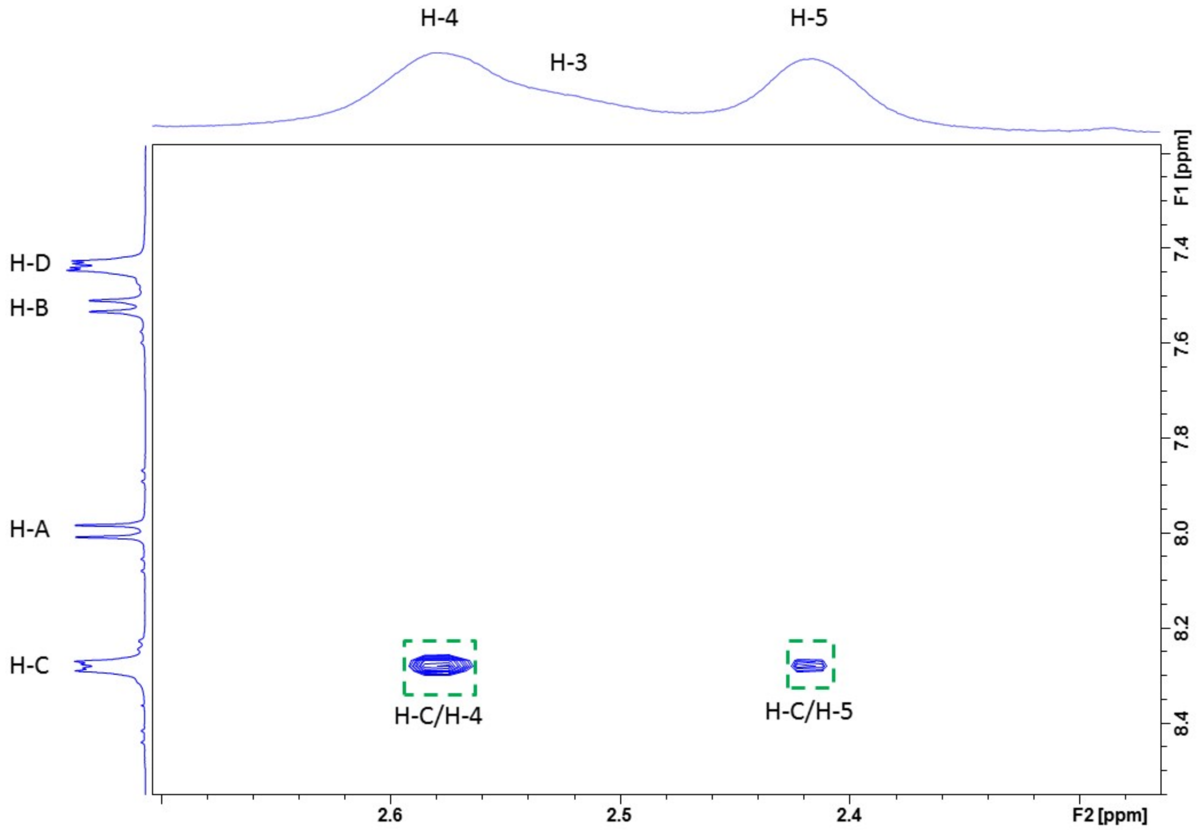

Figure S12. 500 MHz NOESY spectrum of an equimolar mixture of MeβCeB and **1** (30 mM each) in D_2O at (pD 11) 298 K. Medium and weak intermolecular NOE cross peaks are indicated by dashed and dotted squares respectively.

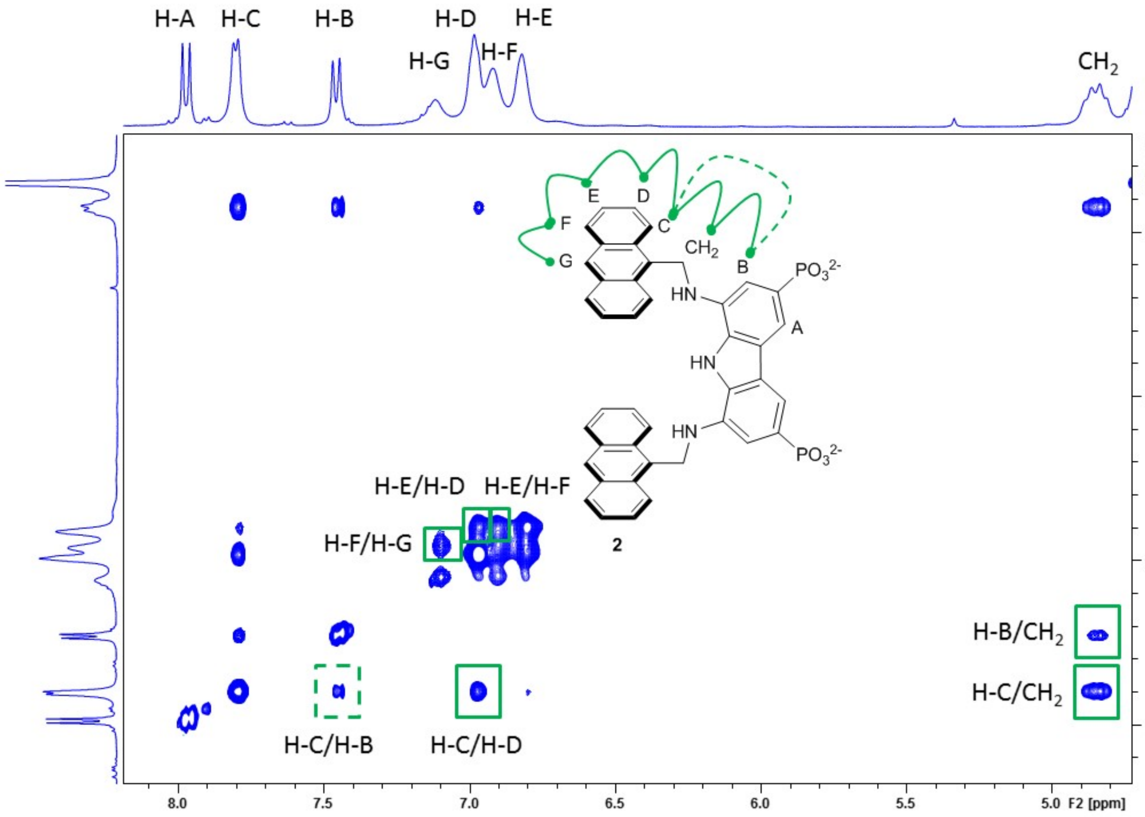

Figure S13. 500 MHz NOESY spectrum of an equimolar mixture of MeβCeB and **2** (20 mM each) in D_2O at 298 K. Intramolecular NOE cross peaks of the receptor **2** are indicated by squares and schematically represented (solid and dashed squares and lines were used to indicate strong and medium NOEs).

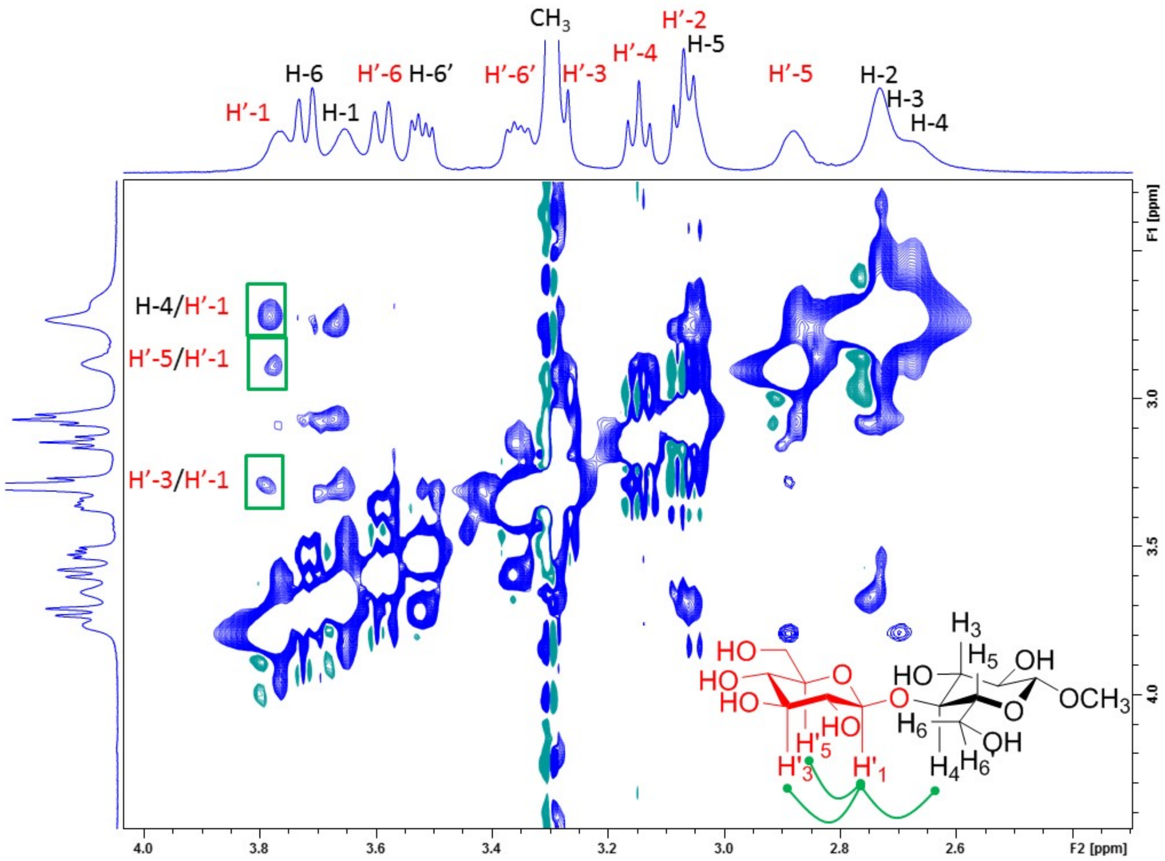

Figure S14. 500 MHz NOESY spectrum of an equimolar mixture of MeβCeB and **2** (20 mM each) in D_2O (pD 11) at 298 K. Intramolecular NOE cross peaks of MeβCeB relating to H'-1 proton are indicated by squares and schematically represented.

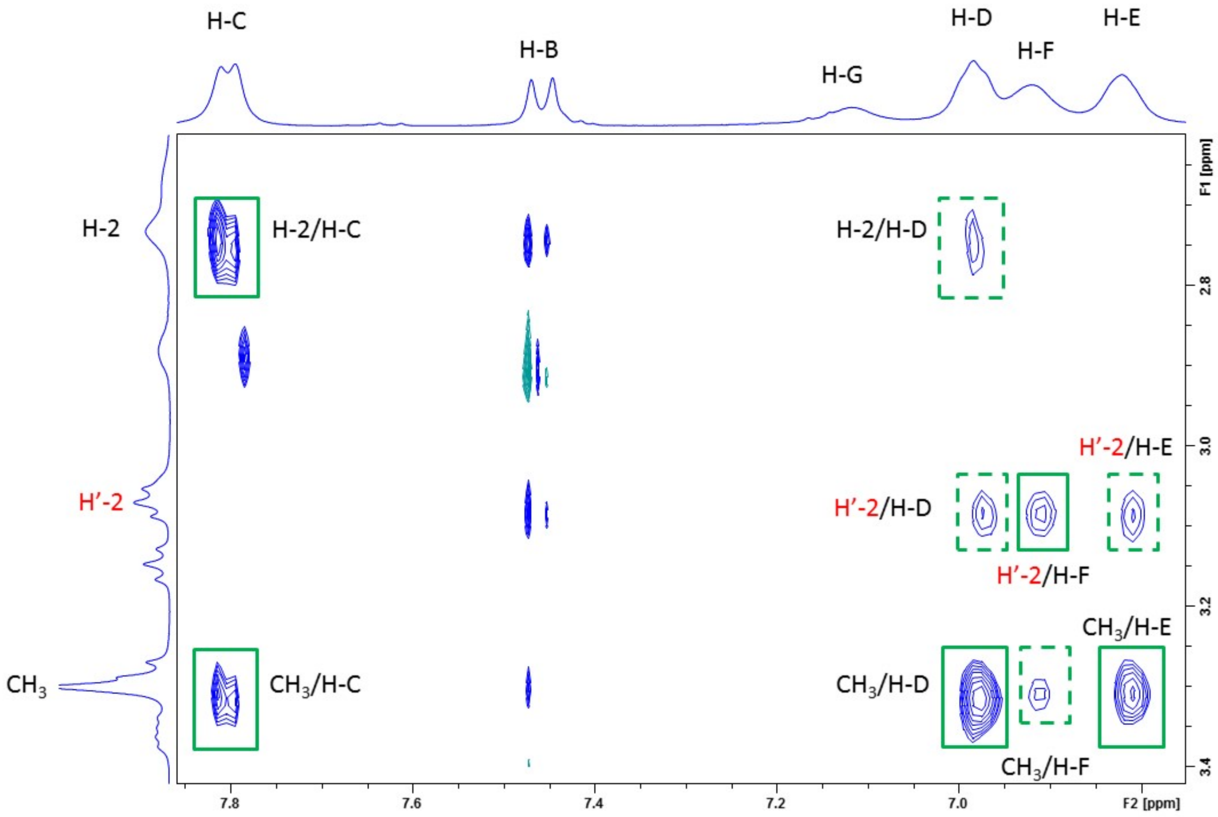

Figure S15. 500 MHz NOESY spectrum of an equimolar mixture of MeβCeB and **2** (20 mM each) in D_2O (pD 11) at 298 K. Strong and medium intermolecular NOE cross peaks are indicated by solid and dashed squares respectively.

Molecular modeling methods. Initial structures of MeβCeB, receptor **1** and receptor **2** were built and minimized using conjugate gradients with the OPLS_2005 force field, water was set as solvent and an extended cutoff was used to treat remote interactions. A maximum number of 5000 iterations were employed with the Polak-Ribiere Conjugate Gradient (PRCG) scheme, until the convergence energy threshold was 0.05. Once the optimum geometries had been achieved, a conformational search protocol was adopted for the receptors, using a Monte Carlo torsional sampling method (MCMM) with automatic setup during the calculation, energy window of 21 kJ mol^{-1}, 10000 maximum number of steps, and 100 steps per torsion of the bond to be rotated. The best structures obtained from this calculation in terms of energy were chosen and then, the disaccharide was manually docked within the receptors cleft with different starting relative orientations and further minimized. Minimization results afford different structures which were employed as input for further conformational search protocols without any constraints. Several complexes were found to be stable, in which the sugar was located inside the receptor cleft. The lowest energy structures were analyzed to check the agreement with experimental NMR data. The protocol returned a family of structures, containing the minimum energy structure of the conformational search, in agreement with the observed NOE data.

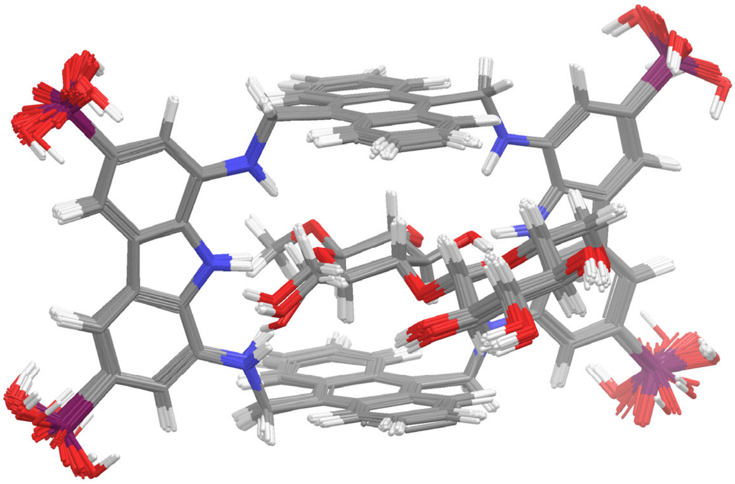

Figure S16. Molecular modelling results from conformational search for the complex of **1** with MeβCeB. Superposition of the 41 energy minimum structures, within an energy window of 5.19 kJ mol^{-1}, identified among the 1152 structures obtained from the calculation.

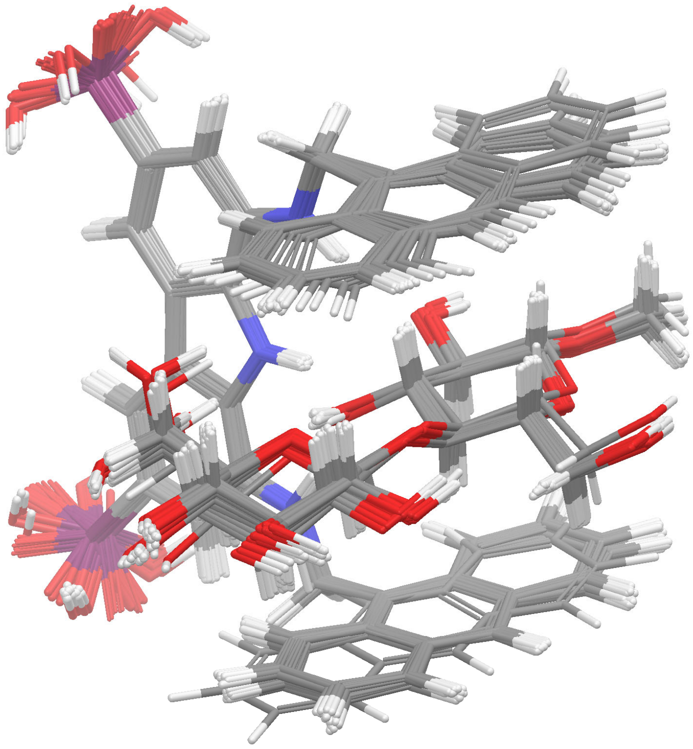

Figure S17. Molecular modelling results from conformational search for the complex of **2** with MeβCeB. Superposition of the 31 energy minimum structures, within an energy window of 8.73 kJ mol^{-1}, identified among the 653 structures obtained from the calculation.

Table S4. Intermolecular NOE cross-peaks found in NOESY spectrum of an equimolar mixture of MeβCeB and **1**, and corresponding distances (Å) obtained from the calculated minimum energy structures.[a]

	H'-1	H'-5	CH_3	H-4	H-5	H-2
H-C			s, 3.16	m, 3.59	m, 3.03	w, 3.69
H-D	s, 2.91	s, 2.43	s, 3.06			-

[a] (s), strong; (m), medium; (w), weak

Table S5. Intermolecular NOE cross-peaks found in NOESY spectrum of an equimolar mixture of MeβCeB and **2**, and corresponding distances (Å) obtained from the calculated minimum energy structures.[a]

	H-2	H'-2	OCH_3
H-C	s, 3.05		s, 4.54
H-D	m, 3.56	m, 3.92	s, 3.17
H-F		s, 3.21	m, 4.02
H-E		m, 2.67	s, 2.79

[a] (s), strong; (m), medium.

Table S6. Distances (Å) measured between MeβCeB protons and the ring planes of receptors anthracenes compliant with CH-π interactions obtained from the minima energy structures of the complexes between MeβCeB and receptors **1** and **2**.

	OCH_3	H-1	H-2	H-3	H-4	H-5	H'-1	H'-2	H'-5
**1**	2.76	2.72	2.63	2.74	2.71	2.71	-	-	-
**2**	-	2.63	2.74	2.74	2.73	2.97	2.83	2.82	2.80

References.

S1. A. Vacca, C. Nativi, M. Cacciarini, R. Pergoli, S. Roelens, *J. Am. Chem. Soc.* **2004**, *126*, 16456-16465.

S2. C. Frassineti, S. Ghelli, P. Gans, A. Sabatini, M. S. Moruzzi, A. Vacca, *Anal. Biochem.* **1995**, *231*, 374-382.
